# Fiber-Dependent Microbiome Glycine Lipids Ameliorate Steatotic Liver Disease in Mice via Mitochondrial Enhancement

**DOI:** 10.1016/j.gastha.2026.101064

**Published:** 2026-07-20

**Authors:** Liya Anto, Lidan Gao, Jaeeun Lee, Chelsea Garcia, Oliver Otoko, Emma Hickey, Neha Mishra, Siyun Kim, Sung Gi Noh, Mi-Bo Kim, Hyunju Kang, Saki Mihori, Saurav Ranjitkar, Alison B. Kohan, Young-Ki Park, Anthony A. Provatas, Clinton Mathias, Oh Sung Kwon, Robert B. Clark, Ji-Young Lee, Frank C. Nichols, Christopher N. Blesso

**Affiliations:** 1 Department of Nutritional Sciences, University of Connecticut, Storrs, Connecticut; 2 Department of Pathobiology and Veterinary Science, University of Connecticut, Storrs, Connecticut; 3 Department of Kinesiology, University of Connecticut, Storrs, Connecticut; 4 School of Food Science and Biotechnology, Kyungpook National University, Daegu, Republic of Korea; 5 Department of Immunology, UConn Health, Farmington, Connecticut; 6 Department of Medicine, Division of Endocrinology and Metabolism, University of Pittsburgh, Pittsburgh, Pennsylvania; 7 Center for Environmental Sciences and Engineering, University of Connecticut, Storrs, Connecticut; 8 Department of Medicine, UConn Health, Farmington, Connecticut; 9 Department of Periodontology, UConn Health, Farmington, Connecticut

**Keywords:** Bacteria, Nutrition, Inflammation

## Abstract

**Background and Aims:**

Oral and gut *Bacteroidota* species produce bioactive glycine lipids (GL), including the core Lipid 342, serine-glycine Lipid 654 (L654), and complex Lipid 1256 (L1256) classes. These microbiome-derived molecules are emerging modulators of host metabolism, but their dietary regulation, systemic presence, and therapeutic potential in metabolic dysfunction–associated steatotic liver disease (MASLD) remain unclear.

**Methods:**

Microbiome-derived GLs were quantified in murine systemic and portal blood, intestinal lymph, and in mouse and human liver using targeted mass spectrometry. Their biological effects were assessed in mouse and cell culture models of diet-induced liver disease following treatment with L654- and L1256-enriched lipid extracts.

**Results:**

L654 and L1256 species were detected in mouse serum, portal plasma, intestinal lymph, and liver, as well as in human liver tissue. A Western-type high-fat diet lowered fecal and hepatic microbiome GLs, whereas fermentable fiber supplementation restored fecal levels (∼7-fold increase), with hepatic GL content showing an inverse association with liver triglycerides. In a 22-week diet-induced MASLD model, administration of L654- or L1256-enriched lipids for 8 weeks significantly reduced hepatic steatosis and histopathological progression. Treatments enhanced mitochondrial DNA content and metabolic function in liver and HepG2 cells. L1256 increased hepatic triggering receptor expressed on myeloid cells 2 expression, associated with lipid sensing and MASLD protection, while L654 modulated splenic cluster of differentiation 4^+^ (CD4^+^) effector memory T cells.

**Conclusion:**

Microbiome-derived GLs are diet-sensitive molecules that protect against MASLD via mitochondrial enhancement, revealing a novel nutritional–microbial axis for liver disease intervention.

## Introduction

Metabolic dysfunction–associated steatotic liver disease (MASLD) is the most common liver disease worldwide, present in over 30% of adults globally.^1^ Approximately 16% of people with MASLD will progress to metabolic dysfunction–associated steatohepatitis (MASH), which is characterized by steatosis, inflammation, and fibrosis.^1^ Current therapeutic options remain limited, highlighting an urgent need for novel treatment strategies. The role of diet in the pathogenesis and management of MASLD is well-established.^2^ However, the contributions of the gut microbiome and its metabolites in the development of MASLD have only recently begun to be widely investigated.

The gut microbiome influences host metabolism through the production of bioactive metabolites. Members of the *Bacteroidota* phylum, which comprise up to 60% of the human gut microbiome, are known for their ability to break down complex glycans and generate fermentation products for the host.^3^ In addition to their glycan metabolism, *Bacteroidota* also produce a unique spectrum of bioactive lipids, including sphingolipids^4^ and glycine lipids (GLs).^5^

Bacterial GLs were first identified in *Flavobacterium* species,^6^^,^^7^ further characterized from the oral pathogen *Porphyromonas gingivalis*,^8^ and later found to be broadly expressed in members of the *Bacteroidota* phylum.^5^^,^^8^, ^9^, ^10^, ^11^, ^12^, ^13^ *Bacteroidota* contain GLs in their cell membranes and shed them in outer membrane vesicles.^12^ The microbiome GL classes identified thus far include the glycine amino lipids, Lipid 342 (L342) and Lipid 567 (L567), named for their negative ion masses, as well as the serine-glycine lipodipeptides, Lipid 430 (L430), Lipid 654 (L654), and Lipid 1256 (L1256).^5^^,^^8^^,^^9^ These lipid classes were named for their dominant ion species but are found alongside minor species variants with different fatty acid substitutions. Of these, L654 and L1256 classes are the major forms detected in most biological samples.

We previously reported that feeding a high-fat diet (HFD) reduces microbiome GLs in mouse feces, serum, and liver tissues.^14^ Prior research has also shown that microbiome GLs activate Toll-like receptor 2 (TLR2) in vitro and in mice.^5^^,^^8^^,^^9^^,^^15^ Despite signaling through TLR2, we have previously found that chronic administration of microbiome GLs attenuates atherosclerosis,^14^ liver injury,^14^ and autoimmune disease^16^ in mouse models. While short-term exposure to GLs can induce cytokine secretion in monocytes^5^ and macrophages^9^ via TLR2, chronic exposure induces TLR2 tolerance^16^^,^^17^ and cross-tolerance to TLR4.^14^

Previously, chronic intraperitoneal administration of L654- and L567-enriched lipid extracts was shown to be hepatoprotective in mouse models of atherosclerosis.^14^ However, critical knowledge gaps persist regarding their therapeutic potential in liver disease models. Additionally, the mechanisms by which these compounds influence hepatic metabolism and the extent to which dietary interventions can modulate their production remain unexplored. The current study was designed to address these knowledge gaps.

## Methods

### Microbiome Glycine Lipid Extraction, High-Performance Liquid Chromatography Fractionation, and Liquid Chromatography-Mass Spectrometry Analysis

Microbiome GL-enriched fractions (L1256 and L654 class species) were extracted from the *Bacteroidota* member *P gingivalis* (ATCC 33277, type strain). While these lipids are found in gut *Bacteroidota*, our team has established robust purification methods using the oral bacterium, *P gingivalis*. Centrifuged bacterial samples were stored as lyophilized pellets until lipid extraction. Each bacterial sample was extracted using the procedure of Bligh and Dyer as modified by Garbus.^18^ The total lipids were fractionated by semipreparative high-performance liquid chromatography (HPLC) using neutral HPLC solvent, pooled, then refractionated using a normal phase HPLC column but with solvent supplemented with 0.1% acetic acid, as previously described.^5^

Fractions were evaluated by liquid chromatography-mass spectrometry (LC-MS) and fractions containing either L1256 or L654 species were pooled by lipid class, and sample purity was evaluated using LC-MS as previously described.^14^ LC-MS analysis of the L654- (Supplementary Figure 2) and L1256-enriched (Supplementary Figure 3) preparations confirmed they were composed predominantly of L654 and L1256 lipid species, respectively. The L654 preparation contained lower abundances of minor species, including L567, while the L1256 preparation contained some deesterified species. Analysis of lipid samples utilized a Sciex500 LC-MS instrument located in the Center for Environmental Sciences and Engineering at the University of Connecticut. The detection and quantification of serine/GLs and surrogate internal standard (D_9_-3-OH-C17:0) used negative ion ESI-MS/MS mode (multiple reaction monitoring [MRM]) as previously described.^5^^,^^9^ Each lipid class was identified based on characteristic retention times of purified or synthetic standards (see Supplementary Table 6), by either quantifying the characteristic molecular parent ion for each lipid class or by quantifying the dominant MS/MS transition ion characteristic of each serine/GL class (MRM) (Supplementary Table 7).

Sonicated lipid preparations were used in experiments, with vehicle CTL solutions undergoing the same sonication procedures as bacterial lipid solutions, as described previously.^14^

### Animals and Diets

Male C57BL/6J and *Ldlr*^−/−^ mice (aged 6 weeks) were obtained from Jackson Laboratory (Bar Harbor, ME) and allowed to acclimate for 2 weeks prior to the start of experiments. Three independent mouse studies were conducted.

In a diet intervention study, we determined the role of dietary fiber in modulating microbiome GL levels. Male *Ldlr*^−/−^ mice (C57BL/6J background) were fed a standard low-fat chow diet (Inotiv Teklad 2918 irradiated diet) (Chow), a Western-type HFD at 5% w/w cellulose, or the same HFD but lacking cellulose and supplemented with fermentable fiber (HFD + fiber) for 8 weeks. Fiber supplementation consisted of a mixture of 5% w/w citrus peel pectin (mainly soluble fiber) (P9135, Sigma-Aldrich) and 5% w/w pea fiber (insoluble and soluble fiber) (HYPF-B3, Jianyuan) for a total of ∼7.5% w/w total dietary fiber and ∼5% w/w soluble fiber. Compositions of Western-type HFD and HFD + fiber are shown in Supplementary Table 1.

In a MASH efficacy study, the effects of chronic microbiome GL administration were examined in a mouse model of diet-induced MASH. Male C57BL/6J mice were fed a HF/HS/HC diet containing 35% w/w fat, 35% w/w sucrose, and 2% w/w cholesterol for 14 weeks to induce liver disease (Supplementary Table 8). Mice were fed HF/HS/HC diets for another 8 weeks while being intraperitoneally injected with bacterial lipid extracts enriched in L654 species (1 μg) (L654), L1256 species (1 μg) (L1256) or vehicle CTL every 48 h. The CTL group received injections of sonicated vehicle (saline with 0.03% v/v ethanol), while the other 2 groups received the injections of 1 μg of the respective sonicated GL preparations (L654 or L1256). This chronic dosing regimen was chosen based on previous research studies administering L654 to mice.^8^^,^^14^

In a GL immune response study, the effects of chronic L654 lipid class administration on T cells were studied in mice. Male C57BL/6J mice were fed for 6 weeks with one of the following dietary regimens: (1) a standard low-fat chow diet (Inotiv Teklad 2918 irradiated diet) (Chow); (2) the HF/HS/HC diet (HFD); (3) the HF/HS/HC diet with vehicle CTL injections 3 times weekly (HFD-Veh); or (4) the HF/HS/HC diet with intraperitoneal injections of bacterial lipid extracts enriched in L654 species (1 μg) administered 3 times weekly (HFD-L654).

Following their respective diets and treatments, the mice were fasted for 6 to 8 h and anesthetized with a ketamine/xylazine cocktail (100 and 10 mg/kg, respectively). Terminal blood was then collected via cardiac puncture for serum isolation, followed by euthanasia. Animals were perfused with sterile saline to clear any residual blood from tissues, which may interfere with analyses. Fecal samples were collected and snap-frozen before storage at −20 °C. Tissues were harvested, weighed, snap-frozen in liquid nitrogen and then stored at −80 °C. Saline-perfused liver sections (left lateral lobe) were fixed in 10% neutral-buffered formalin for 48 h. All mice were housed in a temperature-controlled room and maintained in a 14-h light/10-h dark cycle at the University of Connecticut-Storrs vivarium. All procedures proposed in this study have been approved by the Animal Care and Use Committee of the University of Connecticut-Storrs. All animal experiments were in accordance with the Guide for the Care and Use of Laboratory Animals published by the National Institutes of Health.^19^

### Indirect Calorimetry and Body Composition

After 4 weeks of injections with either the respective vehicle CTL or bacterial lipid extract treatments, the diet-induced MASH mice were individually housed in the Oxymax Comprehensive Lab Animal Monitoring System (Columbus Instruments) to measure activity and energy expenditure, as previously described.^20^ Following a 48-h acclimation period in the Comprehensive Lab Animal Monitoring System, the mice received an additional injection of the respective CTL or treatments. Body composition was assessed using Echo-MRI (Echo Medical Systems) to determine total lean body mass, total fat mass, total body water, and free body water.

### Portal Blood and Intestinal Lymph Collection

Prior to portal blood and mesenteric lymph collection, C57BL/6J mice (aged 8–12 weeks old) were provided standard chow and water ad libitum. Portal blood was collected using ethylenediaminetetraacetic acid-coated syringes from mice anesthetized with ketamine/xylazine. For mesenteric lymph collection, mice were placed under isoflurane anesthesia (induction at 5%, maintenance at 2%), and mesenteric lymph duct cannulas were surgically implanted. Lymph was collected on ice, as previously described.^21^ All surgical procedures were approved by the University of Pittsburgh Internal Animal Care and Use Committee and comply with the NIH Guide for the Care and Use of Laboratory Animals.

### Liver and Splenic Lymphocyte Isolation and Flow Cytometry

Spleen cells were isolated, as previously described,^22^ and resuspended in staining medium (Hanks' Balanced Salt Solution/4-(2-hydroxyethyl)-1-piperazineethanesulfonic acid containing 2% fetal calf serum). Hepatic lymphocytes were isolated from murine liver tissue through mechanical dissociation followed by density gradient centrifugation, as adapted from established protocols.^23^ At sacrifice, livers were perfused with sterile saline to eliminate circulating peripheral blood lymphocytes. Liver lymphocytes were isolated by mechanically dissociating excised tissue through a 100 μm mesh, followed by density-gradient enrichment using 35% Percoll/Heparin and subsequent red blood cell lysis with ACK buffer. The final suspension yielded >2.5 × 10^6^ viable lymphocytes per liver.

Phenotypic characterization of lymphocyte populations was performed using multicolor flow cytometry. Cells were incubated with fluorescently conjugated monoclonal antibodies targeting CD3 (pan-T cell marker), CD4 (helper T cells), CD8 (cytotoxic T cells), CD44 (activation/memory marker), and CD69 (early activation antigen) (BioLegend), following manufacturer-recommended titration protocols validated against positive and negative CTLs. Stained cell suspensions were analyzed using a BD FACSymphony A5 SE flow cytometer at the UConn Health Center Flow Cytometry Core Facility. Data acquisition employed BD FACSDiva software (version 8.0.1) using a standardized gating strategy.

### Serum Biochemical Analysis

Total serum cholesterol, high-density lipoprotein cholesterol, and TG were measured using assay kits from Wako Diagnostics (Richmond, VA). Alanine aminotransferase levels were quantified according to the manufacturer's instructions (Pointe Scientific, Inc; Canton, MI). Paraoxonase 1 lactonase activity was assessed in serum using previously described methods.^24^ Soluble TLR2 and C-C motif chemokine ligand 2 were measured in serum via indirect sandwich ELISAs (R&D Systems; Minneapolis, MN).

### Liver RNA Isolation, Complementary DNA Synthesis, and Real-Time Quantitative Reverse Transcriptase Polymerase Chain Reaction

Total RNA was isolated from liver tissue using TRIzol (Life Technologies, Carlsbad, CA), treated with DNase I and reverse-transcribed into complementary DNA (cDNA) using the iScript cDNA Synthesis Kit (Bio-Rad, Hercules, CA). Real-time quantitative reverse transcriptase polymerase chain reaction was performed with iTaq Universal SYBR Green Supermix (Bio-Rad) on a CFX96 Real-Time PCR Detection System (Bio-Rad). Gene expression was normalized to the geometric mean of the reference genes *Gapdh* (glyceraldehyde 3-phosphate dehydrogenase) and *Rplp0* (ribosomal protein, large, P0) using the 2^−ΔΔCt^ method. Primer sequences are provided in Supplementary Table 9.

### Liver Lipid Extraction and Tissue Histology

Liver lipids were extracted with a modified Folch method as previously described.^14^ Following extraction and solubilization in 1% Triton X-100, total cholesterol, free cholesterol, and TG were measured using enzymatic kits (Wako Diagnostics; Richmond, VA). Cholesteryl esters were also calculated as (total cholesterol – free cholesterol) × 1.67.

For liver histology, fresh tissue sections were fixed in 10% neutral buffered formalin before being processed and stained with hematoxylin and eosin at the Connecticut Veterinary Medical Diagnostic Laboratory in Storrs, CT, as previously reported.^14^ The histopathological scoring for lipid vacuolation (0–5), ballooning (0–5), portal inflammation (0–5), lobular inflammation (0–5), and fibrosis (0–5) were done by a American College of Veterinary Pathologists board-certified pathologist blinded to treatment using slightly modified techniques reported previously.^25^^,^^26^ Liver sections were also stained with Picrosirius Red, as previously described.^27^ The Picrosirius Red–positive area of the liver sections was calculated using ImageJ (National Institutes of Health) image analysis software. Representative photomicrographs were captured for each treatment group.

### Microbiome Glycine Lipid Treatment of HepG2 Cells and Mitochondrial Functional Analysis

Human HepG2 cells were obtained from ATCC (Manassas, VA) and used as an in vitro model of hepatocytes. Cells were cultured in a humidified incubator at 37 °C and 5% CO_2_ and maintained in low-glucose Dulbecco's modified Eagle medium (1 g/L glucose) containing sodium pyruvate, 10% fetal bovine serum (Hyclone, Logan, UT), 2 mM L-glutamine, 100 U/mL penicillin, and 100 μg/mL streptomycin antibiotic (ThermoFisher Scientific, Waltham, MA). HepG2 cells were treated with bacterial lipid extract preparations enriched in L654 (0.654 μg/mL, 6.54 μg/mL) or L1256 (1.256 μg/mL, 12.56 μg/mL), corresponding to approximately equimolar concentrations (∼1 μM and ∼10 μM) of the dominant lipid species (ie, L654 and L1256, respectively), or vehicle CTL every 24 h for a total of 72 h and then were plated into 24-well plates in serum-free media for mitochondrial functional analysis. Cells were subjected to a Mito Stress test using an XFe24 Extracellular Flux Analyzer (Seahorse Biosciences, North Billerica, MA), as described previously.^28^ After completing the assay, total DNA was extracted from each well using NucleoSpin Tissue kit (Macherey-Nagel Inc) to normalize the results.

### Mitochondrial DNA Copy Number

Total DNA was extracted from liver tissue and cells, and the mtDNA copy number was determined by quantitative PCR using a SYBR Green method on a Bio-Rad CFX96 Real-Time system. For this analysis, 16S ribosomal RNA, cytochrome b, and nicotinamide adenine dinucleotide dehydrogenase 1 were mtDNA markers, while 18S ribosomal RNA, hexokinase-2, and lipoprotein lipase served as nuclear reference genes. Gene primers for mtDNA analysis are provided in Supplementary Table 10.

### Quantification of Microbiome Glycine Lipids in Mouse and Human Samples

Liquid nitrogen-preserved samples of human primary liver cancer (liver tumor) and paired adjacent nontumor liver tissues (liver nontumor) were obtained from the UConn Health Center Research Tissue Registry/Repository (Biorepository) (Farmington, CT). Mouse serum, liver tissue, and fecal samples were collected and snap-frozen in liquid nitrogen at the time of sacrifice. Internal standard (D_9_-3-OH-C17:0) was added to samples (serum, plasma, lymph, liver, feces) and lipids were extracted using a modified Bligh and Dyer method and analyzed by ultra-performance liquid chromatography-quadrupole time of flight LC-MS (Sciex500) and MRM as described above and previously.^14^

### Fecal *Bacteroidota* Relative Abundance

DNA was isolated from homogenized mouse fecal pellets using QIAamp Fast DNA Stool Mini kit (Qiagen) according to the manufacturer’s instructions. Relative abundance of *Bacteroidota* was determined by quantitative PCR using 16S rRNA gene–targeted universal (926F and 1062R) and *Bacteroidota*-specific primers (Bac960F and Bac1100R) following the methods of Yang et al.^29^

### Statistical Analysis

Statistical significance was determined using one-way analysis of variance with Fisher Least Squares Difference test for multiple comparisons. The Kruskal-Wallis nonparametric test was applied to nonnormally distributed data. Spearman rank correlation was used to assess relationships between variables. A *P* value < .05 was considered statistically significant. All statistical analyses were conducted using GraphPad Prism (version 8) software, and data are presented as mean ± standard error of the mean. All authors had access to the study data and had reviewed and approved the final manuscript.

## Results

### Detection of Microbiome Glycine Lipids in Systemic Blood, Portal Blood, Intestinal Lymph, and Liver

**Figure 1:**
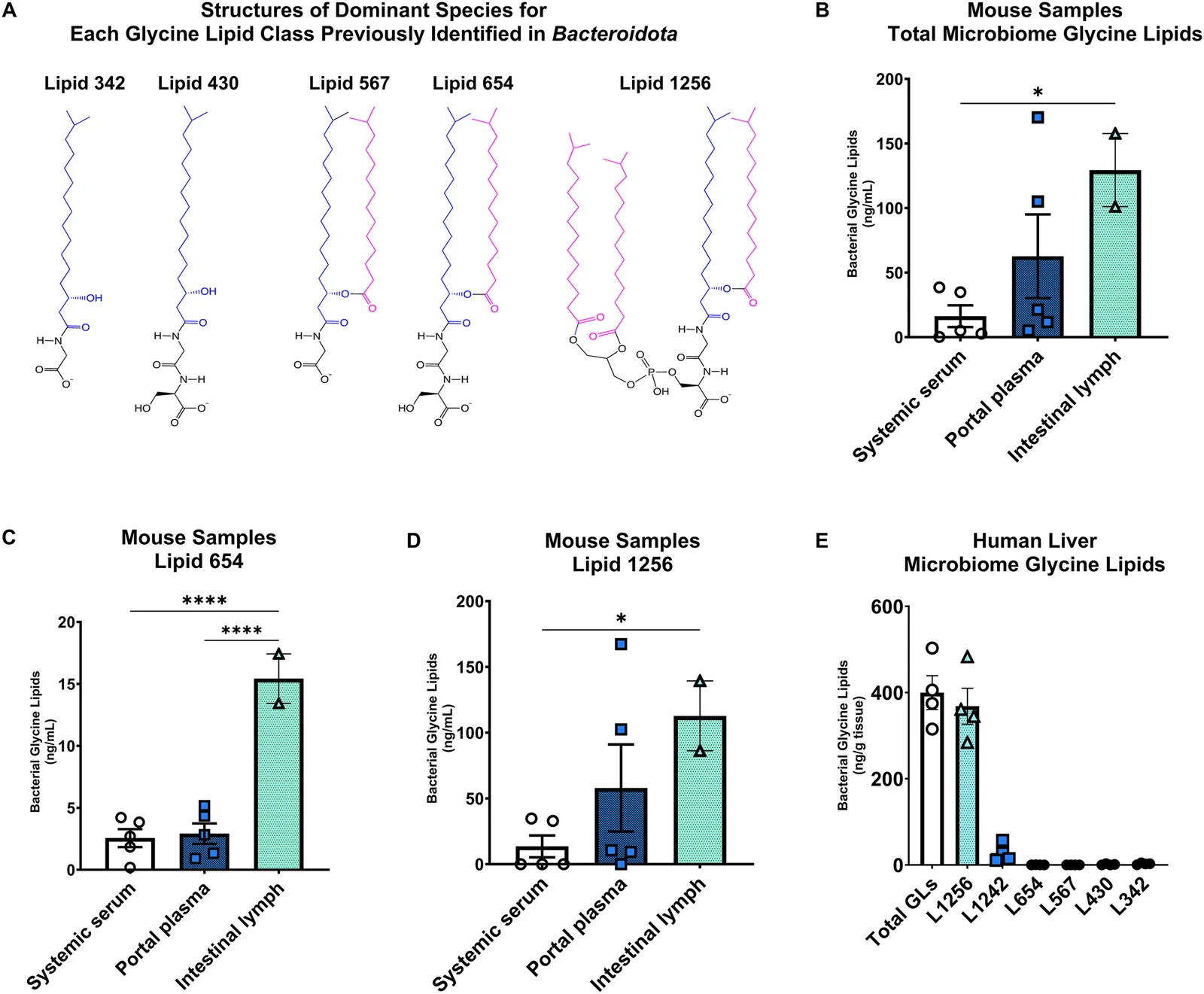
Detection and quantification of microbiome glycine lipids. Structures of microbiome glycine lipids found in *Bacteroidota* depicting the dominant lipid species within each lipid class (A). Note C15:0 (pink) and 3-OH iso C17:0 fatty acids (blue) are present in many of these lipids. Microbiome glycine lipids were quantified by UPLC-MS/MS as follows: total bacterial glycine lipids (B), L654 (C), and L1256 (D) were summed for each lipid class and analyzed across three mouse sample types: systemic circulation serum, portal vein plasma, and intestinal lymph. Values are mean ± standard error of the mean for systemic serum (*n* = 5). Portal plasma (*n* = 5) and intestinal lymph (*n* = 2) glycine lipids were quantified after pooling samples from 3 individual animals. Microbiome glycine lipids in human liver tissues (E) were quantified as ng/g total mass. Values are mean ± standard error of the mean (*n* = 4). Statistical significance determined by one-way ANOVA with Fisher LSD (∗*P* < .05, ∗∗∗∗*P* < .0001). ANOVA, analysis of variance; LSD, Least Squares Difference; UPLC-MS/MS, ultra-performance liquid chromatography-tandem mass spectrometry.

Previous work from our group identified microbiome GLs as normal constituents of host feces, with detectable levels in serum and tissues of mice and humans.^11^^,^^14^^,^^30^ The structures of the dominant lipid species of the GL classes are shown in Figure 1A. To investigate their potential absorption pathways, we quantified these lipids in portal vein plasma, systemic serum, and small intestinal lymph of C57BL/6J mice. Microbial GLs were found in all compartments, with the highest concentrations in intestinal lymph. (Figure 1B–D). The microbiome GLs, particularly L1256 class species, were also detected in human hepatic tissue (Figure 1E), representing the first demonstration that these microbiome GLs are present in human liver.

### Fermentable Fiber Is a Major Dietary Factor Regulating Gut Microbiome Glycine Lipids

We previously reported decreases in fecal, liver, and serum microbiome GLs (including L654) in low-density lipoprotein receptor (*Ldlr*)^−/−^ mice when they were switched from a low-fat chow diet to a Western-type HFD (45% kcal fat).^14^ In addition to fat differences, chow (Teklad 2918) contains soluble fibers and ∼15% (w/w) insoluble fiber, while the HFD contains only 5% (w/w) insoluble cellulose.

**Figure 2:**
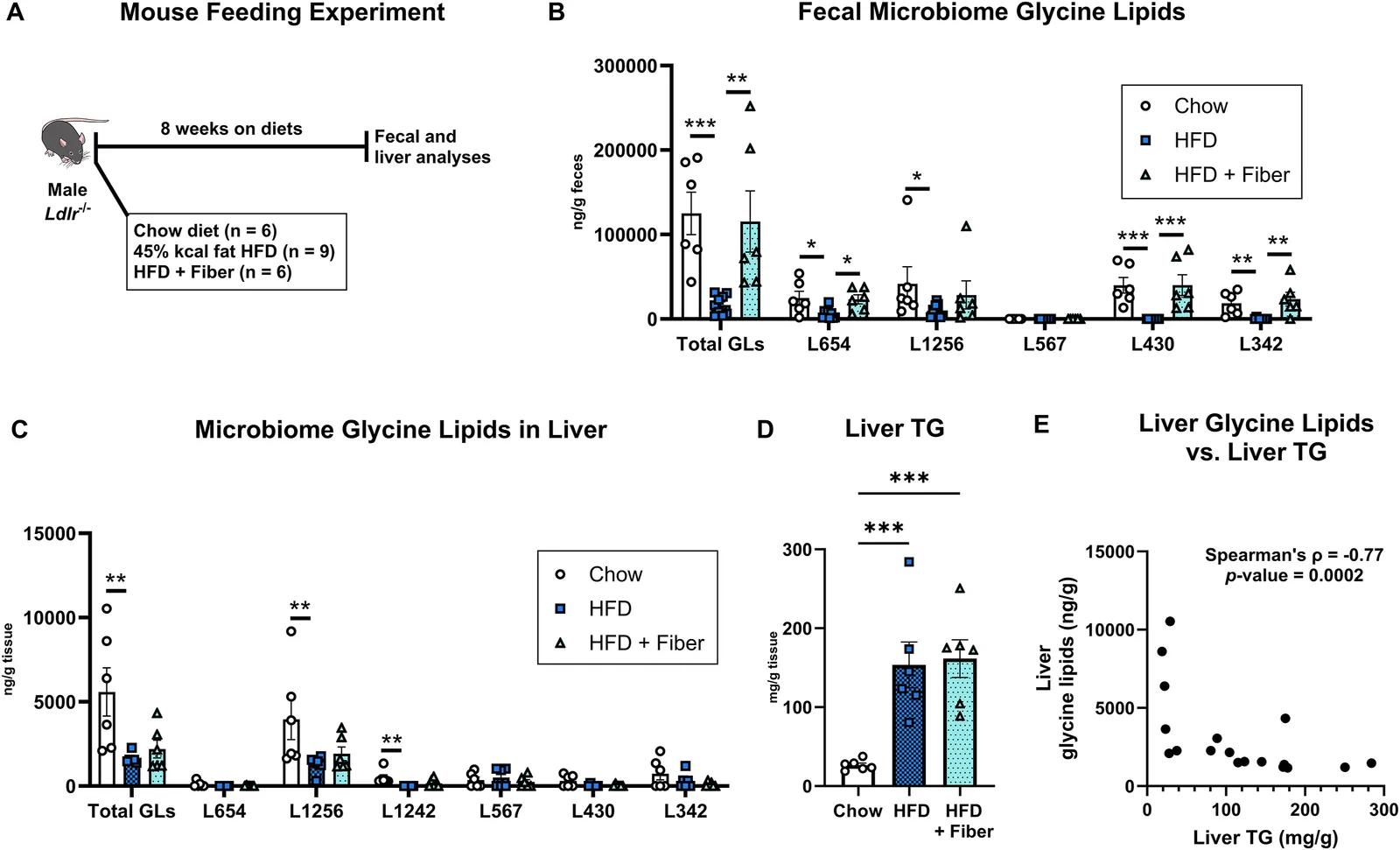
Fermentable fiber is a major dietary factor regulating gut microbiome glycine lipids. Male *Ldlr*^−/−^ mice (C57BL/6J) were fed low-fat chow, Western HFD (5% cellulose), or HFD + fiber (pectin/pea fiber replacing cellulose; ∼7.5% total, ∼5% soluble fiber) for 8 weeks (A). Lipids were extracted and total microbiome glycine lipids (total GLs), L654, L1256, Lipid 1242 (L1242), L567, L430, and L342 in cecal feces (B) and liver (C) lipid extracts were quantified by UPLC-MS/MS (*n* = 6–9, mean ± standard error of the mean). Hepatic triglycerides (TG) (D) and associations between hepatic microbiome glycine lipids and TG via Spearman correlation (E). Statistical significance determined by Kruskal-Wallis nonparametric test (∗*P* < .05, ∗∗*P* < .01, ∗∗∗*P* < .001). Illustration from NIAID NIH BIOART Source (bioart.niaid.nih.gov/bioart/281). UPLC-MS/MS, ultra-performance liquid chromatography-tandem mass spectrometry.

To test whether fermentable fiber rescues microbiome GLs in feces and liver, male *Ldlr*^−/−^ mice (C57BL/6J background) were fed a low-fat chow diet (Chow), a HFD with 5% w/w cellulose, or the same HFD but lacking cellulose and supplemented with fermentable fiber (HFD + fiber) for 8 weeks (Figure 2A). Fiber supplementation (for a total of ∼7.5% w/w dietary fiber and ∼5% w/w soluble fiber) consisted of a mixture of citrus peel pectin (mainly soluble fiber) and pea fiber (insoluble and soluble fiber) (Supplementary Table 1). These fiber types promoted *Bacteroides* growth previously.^31^^,^^32^ Although chow-fed animals consumed more food by weight, terminal body weight, percent body weight change from baseline, and liver weight were generally higher in the HFD-fed groups than in chow-fed controls (CTLs; Supplementary Table 2). HFD reduced fecal microbiome GLs by ∼87% vs chow, including reductions in L654 (−74%), L1256 (−76%), L430 (−100%), and L342 (−98%) species (Figure 2B). The supplementation of HFD with fermentable fiber increased fecal *Bacteroidota* relative abundance (Supplementary Figure 1). Furthermore, fermentable fiber markedly increased the fecal microbiome GLs compared to HFD alone, with ∼7-fold higher total microbiome lipids, ∼3.6-fold more L654, ∼2.9-fold more L1256, ∼79-fold more L342, and detectable levels of L430 species. Despite these fecal recoveries, hepatic microbiome lipids and hepatic triglycerides (TG) remained unchanged with fiber (Figure 2C and D). However, hepatic microbiome GLs were inversely associated with liver triglyceride content (Figure 2E). Thus, these results show these lipids are diet-modifiable and that their accumulation in the liver is relevant to hepatic health.

### Microbiome Glycine Lipids Attenuate Histopathological Features of Liver Disease Progression in a Mouse Model of Metabolic Dysfunction–Associated Steatohepatitis

**Figure 3:**
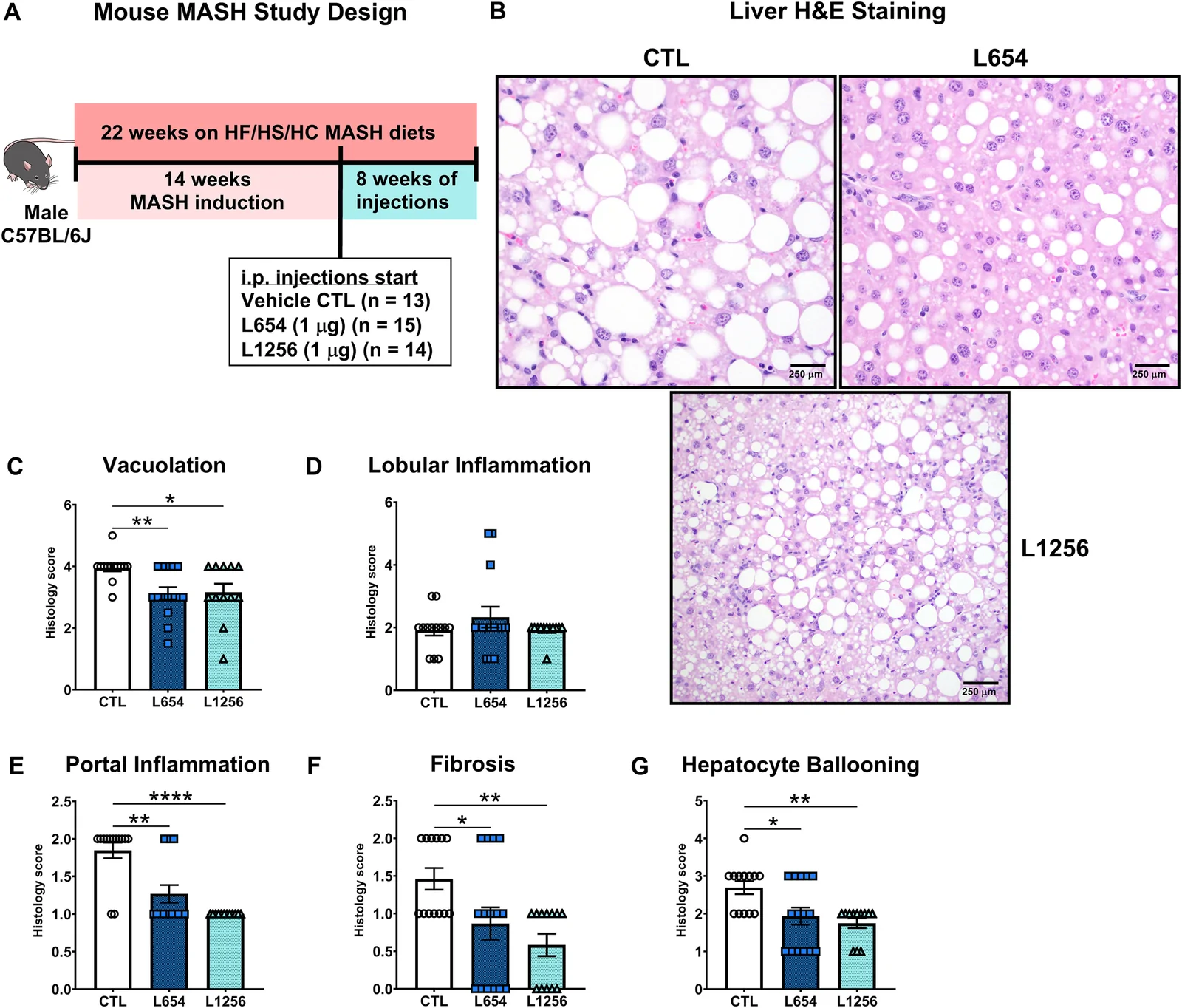
Microbiome glycine lipids attenuate histopathological features of liver disease progression in a mouse model of MASH. The effects of chronic administration of L654, L1256, or vehicle control (CTL) were studied in C57BL/6J mice (A). Liver sections were hematoxylin and eosin stained (B) for visualizing hepatic steatosis (x200). Blinded histopathological scoring of liver sections for vacuolation (C), lobular inflammation (D), portal inflammation (E), fibrosis (F), and hepatocyte ballooning (G) (*n* = 13–15, mean ± standard error of the mean). Statistical significance determined by Kruskal-Wallis nonparametric test (∗*P* < .05, ∗∗*P* < .01, ∗∗∗*P* < .001, ∗∗∗∗*P* < .0001). Illustration from NIAID NIH BIOART Source (bioart.niaid.nih.gov/bioart/281).

Given the presence of L654 and L1256 in portal plasma and liver, we next evaluated their bioactivity in a diet-induced MASH model. Male C57BL/6J mice were fed a high-fat (35% w/w), high-sucrose (35% w/w), and high-cholesterol (2% w/w) diet for 22 weeks to induce MASH. During the final 8 weeks, mice received intraperitoneal injections every 48 h of either vehicle CTL or 1 μg of bacterial lipid extracts enriched in L654 species or L1256 species (Figure 3A). We used a two-step semipreparative HPLC purification method to prepare highly-enriched fractions of L654 (Supplementary Figure 2) and L1256 (Supplementary Figure 3) classes of species and will therefore refer to them as L654 and L1256 treatments, respectively.

After 8 weeks, treatments minimally impacted food intake, body/tissue weights, calorimetry, and wheel-running, though free body water decreased in L654- and L1256-treated mice (Supplementary Table 3, Supplementary Figure 4). Serum analyses revealed L654-mediated reductions in alanine aminotransferase (−23%) and soluble TLR2 (−35%) compared to CTLs (Supplementary Table 4).

Blinded histopathological assessment for MASH was conducted on hematoxylin and eosin-stained livers. Consumption of the high-fat/high-sucrose/high-cholesterol (HF/HS/HC) diet for 22 weeks resulted in substantial MASH development in the CTL group (Figure 3B). Except for lobular inflammation, the histologic scores for lipid vacuolation (steatosis), portal inflammation, fibrosis, and hepatocyte ballooning were each significantly lower in both microbiome GL-treated groups than in CTLs (Figure 3C–G). Although microbiome GLs improved multiple histologic features, they did not significantly affect the fibrotic area quantified in Picrosirius red-stained liver sections (Supplementary Figure 5). Hepatic lipids were reduced by L654 and L1256 treatments, consistent with the histopathological findings (Table 1). Despite these benefits, the levels of microbiome GLs in liver (Table 1) and feces (Supplementary Table 5) did not differ between groups.

**Table 1:** Liver Lipids After 22 Wk of Diet With 8 Wk of Treatments

Variable	Control	L654	L1256
Triglycerides (mg/g)	269.6 ± 40.2	192.2 ± 15.1^a^	193.2 ± 13.5^a^
Cholesterol (mg/g)	21.8 ± 4.25	14.6 ± 1.99	12.3 ± 0.92^a^
Cholesteryl ester (mg/g)	19.0 ± 2.99	12.2 ± 1.38^a^	11.9 ± 0.46^a^
Free cholesterol (mg/g)	10.4 ± 2.59	7.36 ± 1.64	5.13 ± 0.77^b^
Microbiome glycine lipids (ng/g)	1290.8 ± 235.6	1341.8 ± 279.4	1257.8 ± 203.3
Lipid 1256 (ng/g)	781.6 ± 196.8	736.6 ± 229.0	669.5 ± 181.5

Values are mean ± standard error of the mean for triglycerides and cholesterol (*n* = 13–15/group) and microbiome glycine lipids (*n* = 5/group).

a Indicates *P* value <.05 vs control via one-way analysis of variance (ANOVA).

b Indicates *P* value = .05 vs control.

### Microbiome Glycine Lipids Induce Hepatic Messenger RNA Expression of Genes Involved in Fatty Acid Oxidation and Promote Hepatocyte Mitochondrial Function

**Figure 4:**
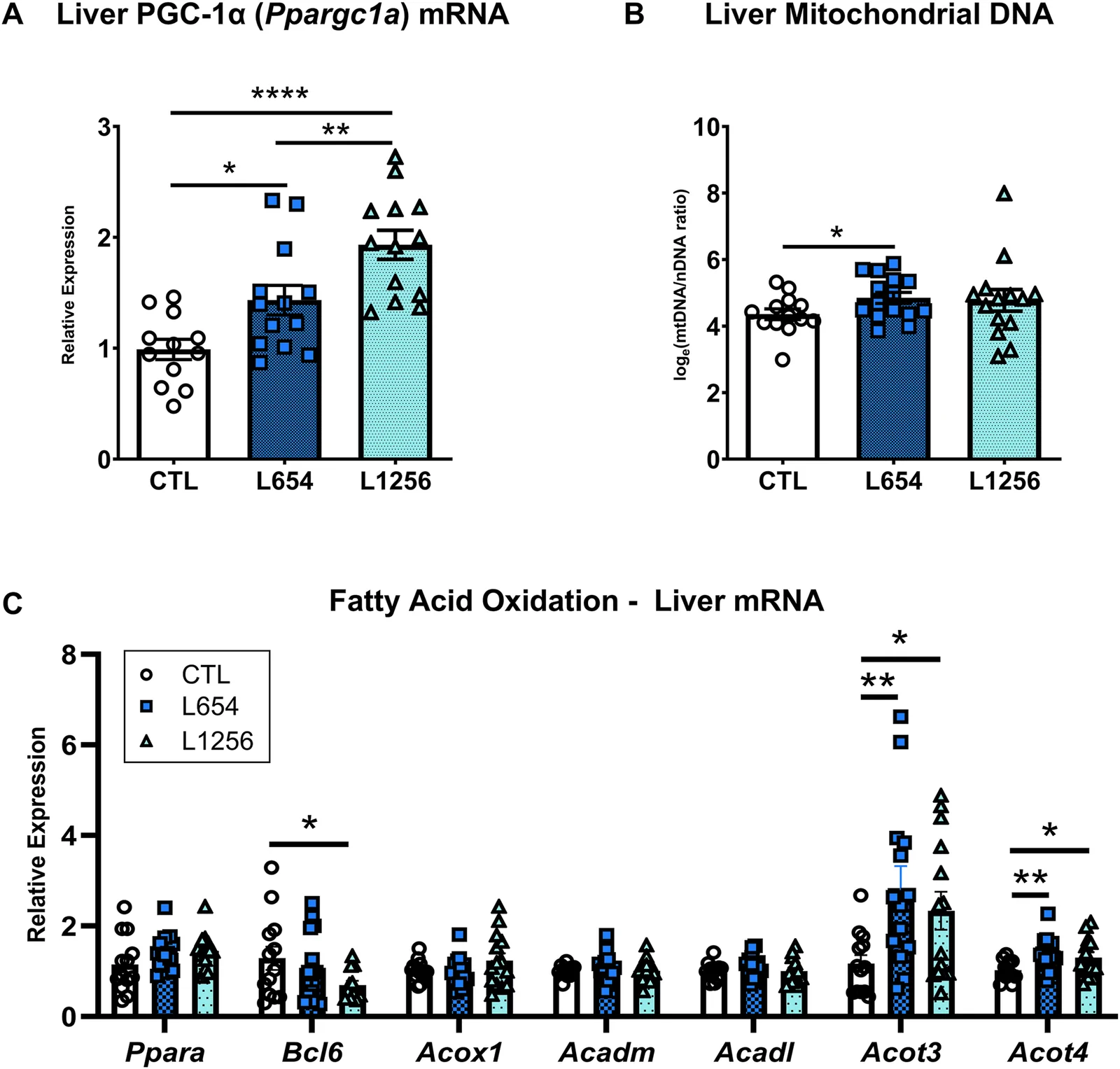
Microbiome glycine lipids induce hepatic mRNA expression of genes involved in fatty acid oxidation and hepatic mitochondrial DNA. Quantification of mRNA expression of PGC-1α (*Ppargc1a*) (A) and fatty acid oxidation genes (C) is shown (*n* = 12–15, mean ± standard error of the mean). Total liver DNA was isolated and mitochondrial DNA copy number (B) quantified by mitochondrial DNA (mtDNA) to nuclear DNA (nDNA) ratios (*n* = 13–15, mean ± standard error of the mean). Ratios were Log_e_ transformed due to positively skewed distributions. Statistical significance determined by one-way ANOVA with Fisher LSD (∗*P* < .05, ∗∗*P* < .01, ∗∗∗∗*P* < .0001). ANOVA, analysis of variance; LSD, Least Squares Difference.

TLR2 signaling has been previously linked to hepatic peroxisome proliferator-activated receptor-γ coactivator 1-α (PGC-1α) induction,^33^^,^^34^ so we next assessed hepatic messenger RNA (mRNA) expression of *Ppargc1a* (PGC-1α) and other genes involved in fatty acid oxidation (Figure 4). *Ppargc1a* (+45–95%) showed increased mRNA expression in L654- and L1256-treated mice (Figure 4A). PGC-1α is a known coactivator of peroxisome proliferator-activated receptor alpha (PPARα).^35^ While *Ppara* (PPARα) mRNA remained unchanged, microbiome GLs regulated several genes governing fatty acid oxidation (Figure 4C). Both treatments upregulated peroxisomal thioesterases *Acot3* (+99–142%) and *Acot4* (+28–37%), involved in fatty acid oxidation regulation.^36^ L1256 downregulated B cell lymphoma 6 mRNA (−46%), which acts as a suppressor of PPARα-regulated target genes, including *Acot3/4* expression.^37^ Additionally, L654 increased hepatic mitochondrial DNA (mtDNA) copy number, as measured by the mitochondrial-to-nuclear DNA ratio (Figure 4B).

**Figure 5:**
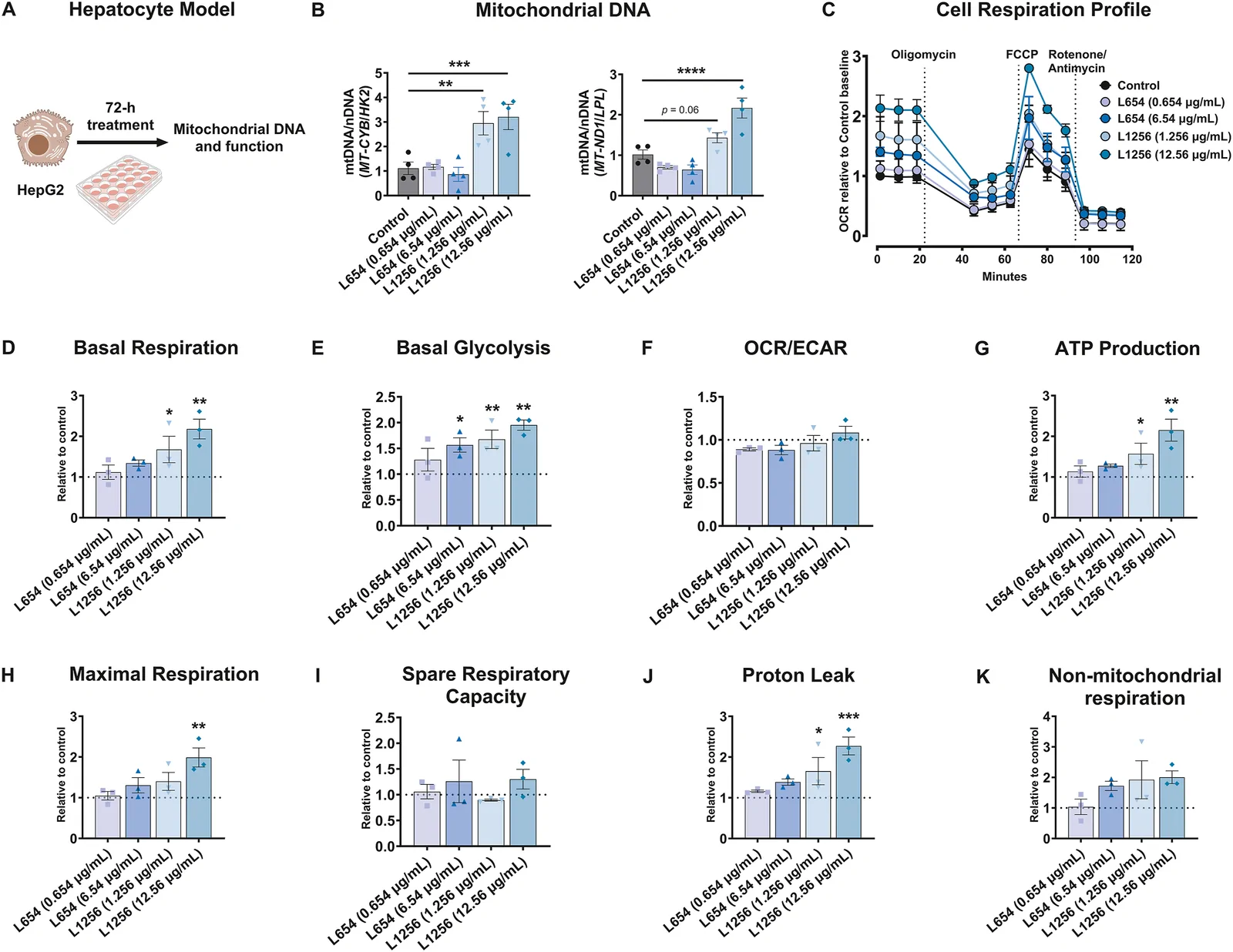
Microbiome-derived glycine lipids enhance mitochondrial bioenergetics in HepG2 cells. Cells were treated with L654, L1256, or vehicle control for 72 h before analysis of mitochondrial DNA and mitochondrial function (A). Mitochondrial DNA copy number (B) quantified by mitochondrial DNA (mtDNA) to nuclear DNA (nDNA) ratios. Results are shown for oxygen consumption rate (OCR) during the Mito Stress test over time (C), basal respiration (D), basal glycolysis (E), OCR/extracellular acidification rate (ECAR) ratio (F), adenosine triphosphate production (G), maximal respiration (H), spare respiratory capacity (I), proton leak (J), and nonmitochondrial respiration (K). Data are normalized to control within each experiment (indicated by dotted line) and presented as mean ± standard error of the mean from three independent experiments. Statistical significance determined by one-way ANOVA with Fisher LSD (∗*P* < .05, ∗∗*P* < .01, ∗∗∗*P* < .001). Illustrations from NIAID NIH BIOART Source (bioart.niaid.nih.gov/bioart/4 and bioart.niaid.nih.gov/bioart/202). ANOVA, analysis of variance; LSD, Least Squares Difference.

To evaluate further the effects of microbiome GLs on the bioenergetics and mitochondrial function of liver cells, we conducted experiments where we treated the human HepG2 cells with either L654 (0.654 μg/mL, 6.54 μg/mL) or L1256 (1.256 μg/mL, 12.56 μg/mL), corresponding to approximately equimolar concentrations (∼1 μM and ∼10 μM) of the dominant lipid species (ie, L654 and L1256, respectively), or vehicle CTL every 24 h for 3 consecutive days (Figure 5A). mtDNA was increased in this model by L1256 but not by L654 (Figure 5B). After 3 days of treatment, cells were subjected to a mitochondrial stress test to evaluate respiratory function. Results from 3 independent experiments were normalized to vehicle CTL and analyzed (Figure 5C–K; representative experiment shown in Supplementary Figure 6). Compared to vehicle CTL, the highest concentration of L1256 treatment significantly increased HepG2 cell basal respiration, glycolysis, adenosine triphosphate production, maximal respiration, and proton leak by approximately 2-fold. No statistically significant changes were observed in oxygen consumption rate/ extracellular acidification rate ratio, spare respiratory capacity, or nonmitochondrial respiration. At the lowest concentration, the L1256 treatment had more modest effects. Thus, L1256 appears to act as metabolic enhancers in hepatocytes, significantly increasing mitochondrial content and function while also enhancing glycolytic capacity.

### Microbiome Glycine Lipids Affect Hepatic mRNA Expression of Genes Involved in Inflammation

**Figure 6:**
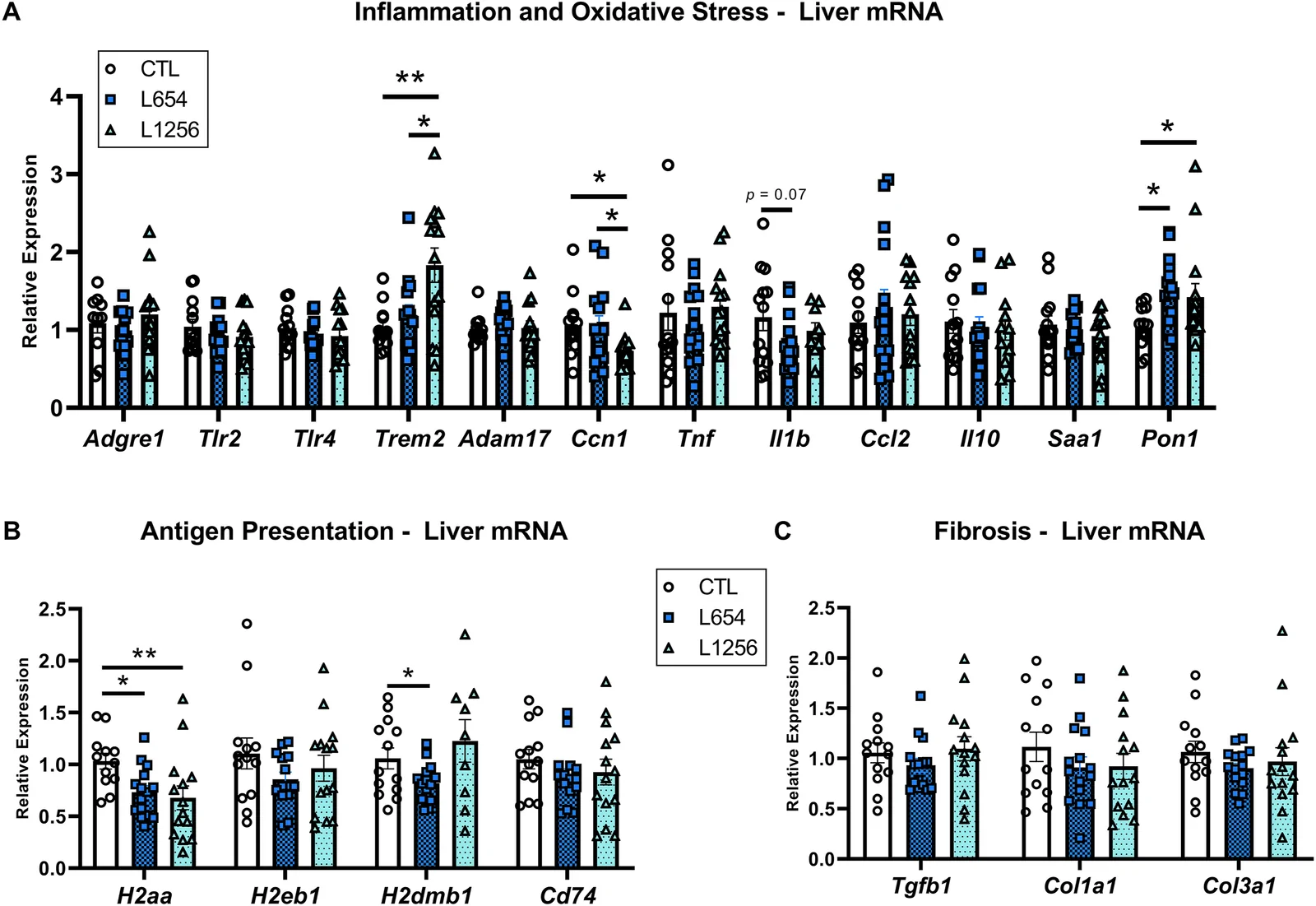
Microbiome glycine lipids affect hepatic mRNA expression of genes involved in inflammation. Gene expression related to inflammation and oxidative stress (A), antigen presentation (B), and fibrosis (C) (*n* = 9–15, mean ± standard error of the mean). Statistical significance determined by one-way ANOVA with Fisher LSD (∗*P* < .05, ∗∗*P* < .01). ANOVA, analysis of variance; LSD, Least Squares Difference.

To assess the impact of microbiome GLs on inflammation and fibrosis, we next analyzed the mRNA expression of a broad panel of genes related to macrophage activity, immune signaling, and fibrogenesis in mouse liver (Figure 6).

The hepatic mRNA expression of triggering receptor expressed on myeloid cells 2 (*Trem2*), which encodes for TREM2, was upregulated by L1256 (+77%) (Figure 6A). The expression of *Ccn1* mRNA, which is involved in activating TLR2/4 signaling,^38^ decreased by L1256 (−31%). The mRNA expression of the negative acute phase protein, paraoxonase 1, was also increased by both microbiome GLs by 37%–46%. Major histocompatibility complex class II genes *H2-Aa* and *H2-DMb1* were significantly decreased by lipid treatments (Figure 6B). However, no significant changes were observed in other inflammation- or fibrosis-associated genes (Figure 6A–C).

To investigate whether these molecular changes translated to cellular immune adaptations, male C57BL/6J mice underwent four 6-week regimens: (1) chow; (2) HFD (high-fat/high-cholesterol/high-sucrose); (3) HFD + vehicle; or (4) HFD + L654 (1 μg/dose, 3x weekly). Flow cytometry of intrahepatic lymphocytes revealed HFD increased hepatic effector memory cluster of differentiation 4^+^ (CD4^+^) (CD44^+^) and CD8^+^ (CD44^+^CD69^+^) T cells but reduced naïve subsets. While L654 did not reverse hepatic T cell shifts, it reduced splenic effector memory CD4^+^ T cells (CD44^+^CD69^-^) and restored naïve CD4^+^ frequencies (Supplementary Figures 7-9), indicating splenocyte modulation was distinct from its hepatic gene effects.

## Discussion

Microbiome GLs produced by *Bacteroidota* accumulate in host tissues^11^^,^^14^ and undergo cellular hydrolysis, though L1256 degrades slowly enough to promote retention.^39^ We demonstrate these lipids circulate in portal blood and intestinal lymph and that their chronic administration confers hepatoprotection against diet-induced MASH. Moreover, dietary fermentable fiber supplementation restores microbiome GL production during HFD feeding, establishing a novel mechanistic link between dietary fiber, gut microbial metabolism, and liver health.

Importantly, we establish that microbiome GLs upregulate hepatic PGC-1α (*Ppargc1a*) mRNA expression and increase mtDNA content. This represents a potential mechanism underlying the hepatoprotective effects observed with L654 and L1256 in the current study and with L567 and L654 in previous studies.^14^ PGC-1α is a key transcriptional coactivator involved in promoting PPARα-dependent fatty acid oxidation and PPARα-independent mitochondrial biogenesis.^40^ It is known to play a key role in preventing MASLD, and its dysfunction is associated with liver disease pathogenesis.^40^ Whether the regulation of PGC-1α expression and mitochondrial enhancement was linked to TLR signaling or some other mechanism is unclear. Activation of TLR2 signaling has been linked to *Ppargc1a* mRNA expression in the AML12 hepatocyte cell line^33^ and mouse liver.^34^

We also observed that bacterial GL extracts enriched in L1256, but not L654, enhanced mtDNA content and function in HepG2 cells. Increases in cell respiration are unlikely to be due to the GLs providing fatty acid substrate for oxidation, as cells were washed and replaced with fetal bovine serum-free assay buffer prior to respiration testing. Currently, it is unclear whether the differences observed between L654 and L1256 are related to potency in their TLR2 activation/regulation or result from unknown interactions with other receptors. Non-TLR effects of other glycine aminolipids have been previously reported, including activation of PPARα^41^ and G2 accumulation protein/ G protein-coupled receptor 132.^13^ Future studies should elucidate the upstream signaling mediating these effects and their direct impact on lipid metabolism across hepatic cell populations.

To date, most research on microbiome-derived GLs has centered on their immunomodulatory effects. Acute, high-dose exposure to these bacterial GLs can activate pro-inflammatory responses via TLR2/TLR6 heterodimers, which may contribute to pathogenesis in certain disease contexts like periodontal disease.^42^ However, a chronic, low-dose exposure, which is more reflective of physiological gut microbiome exposure, appears to induce tolerance and reduce inflammatory responses.^16^^,^^17^ In the present study, classical inflammatory biomarkers in the liver showed only minor changes at the mRNA level; however, the L1256-enriched extract significantly increased hepatic *Trem2* mRNA expression. This up-regulation links our findings to emerging evidence implicating TREM2 in metabolic liver disease.^43^ TREM2 is present on myeloid cells and can act as a sensor for endogenous and exogenous lipid ligands.^44^ Prior work from Mihori et al^17^^,^^45^ showed that a *Bacteroidota*-targeting antibiotic regimen in mice downregulated *Trem2* expression in splenic monocytes and TREM2 surface expression in peritoneal macrophages, whereas chronic L654 injections restored these measures. Our findings showing selective TREM2 upregulation by L1256 in liver, not L654, suggests a compartment-specific regulation by these lipids. Recent studies have highlighted the protective effects of liver TREM2 related to inhibition of inflammation, promotion of efferocytosis, and improvements in mitochondrial energy metabolism.^46^, ^47^, ^48^ Our observed reduction in MASLD histopathological features, including portal inflammation and hepatocyte ballooning, is consistent with the protective role of TREM2 in liver diseases.

The dramatic responsiveness of microbiome GLs to dietary fiber manipulation establishes these molecules as new targets for dietary interventions. This fiber-dependence likely reflects the polysaccharide utilization capabilities of *Bacteroidota* producers. We chose pea fiber and citrus pectin for this investigation and observed an increase in fecal *Bacteroidota* relative abundance. Further investigation is warranted to determine how other dietary fiber types regulate microbiome GL levels in feces and tissues, as well as to evaluate their broader translational potential and specific effects on liver disease pathology.

Although we revealed diet dependence and hepatic effects of microbiome GLs, limitations remain. While detected in human liver, we did not evaluate whether levels differ in MASLD patients. Additionally, to isolate direct hepatic effects via portal drainage, we utilized intraperitoneal injections.^49^ Because this bypasses potential gastrointestinal interactions, future studies should specifically investigate these local effects. Moving forward, dietary fiber may serve as an ideal intervention to stimulate in situ gut production of GLs during future bioactivity experiments.

## Conclusion

Overall, this work identifies microbiome-derived GLs as a novel class of bioactive molecules that may mediate the hepatoprotective effects of commensal microbes against metabolic liver disease. The ability to modulate these protective molecules through dietary fiber intake offers significant clinical potential for preventing and treating MASLD, particularly given the growing global burden of metabolic liver disease.

## Supplementary Materials

Supplementary Materials

Extended PDF
