# Supplementary material for: Fiber-Dependent Microbiome Glycine Lipids Ameliorate Steatotic Liver Disease in Mice via Mitochondrial Enhancement
Source: Gastro Hep Adv. 2026 Jul 20;5(10):101064. doi: 10.1016/j.gastha.2026.101064 (PMC13499107; doi:10.1016/j.gastha.2026.101064)
Supplement: Supplementary file 1 [file mmc1.pdf]

Supplemental Information:

Fiber-dependent microbiome glycine lipids ameliorate steatotic liver disease in mice via mitochondrial enhancement

Liya Anto[1], Lidan Gao[1], Jaeeun Lee[1], Chelsea Garcia[1], Oliver Otoko[1], Emma Hickey[1], Neha Mishra[2], Siyun Kim[3], Sung Gi Noh[3], Mi-Bo Kim[1], Hyunju Kang[4], Saki Mihori[5], Saurav Ranjitkar[1], Alison B. Kohan[6], Young-Ki Park[1], Anthony A. Provatas[7], Clinton Mathias[1], Oh Sung Kwon[3], Robert B. Clark[5,8], Ji-Young Lee[1], Frank C. Nichols[9], and Christopher N. Blesso[1]

[1]Department of Nutritional Sciences, University of Connecticut, Storrs, CT, USA

[2]Department of Pathobiology and Veterinary Science, University of Connecticut, Storrs, CT, USA

[3]Department of Kinesiology, University of Connecticut, Storrs, CT, USA

[4]School of Food Science and Biotechnology, Kyungpook National University, Daegu, Republic of Korea

[5]Department of Immunology, UConn Health, Farmington, CT, USA

[6]Department of Medicine, Division of Endocrinology and Metabolism, University of Pittsburgh, Pittsburgh, PA, USA

[7]Center for Environmental Sciences and Engineering, University of Connecticut, Storrs, CT, USA

[8]Department of Medicine, UConn Health, Farmington, CT, USA

[9]Department of Periodontology, UConn Health, Farmington, CT, USA

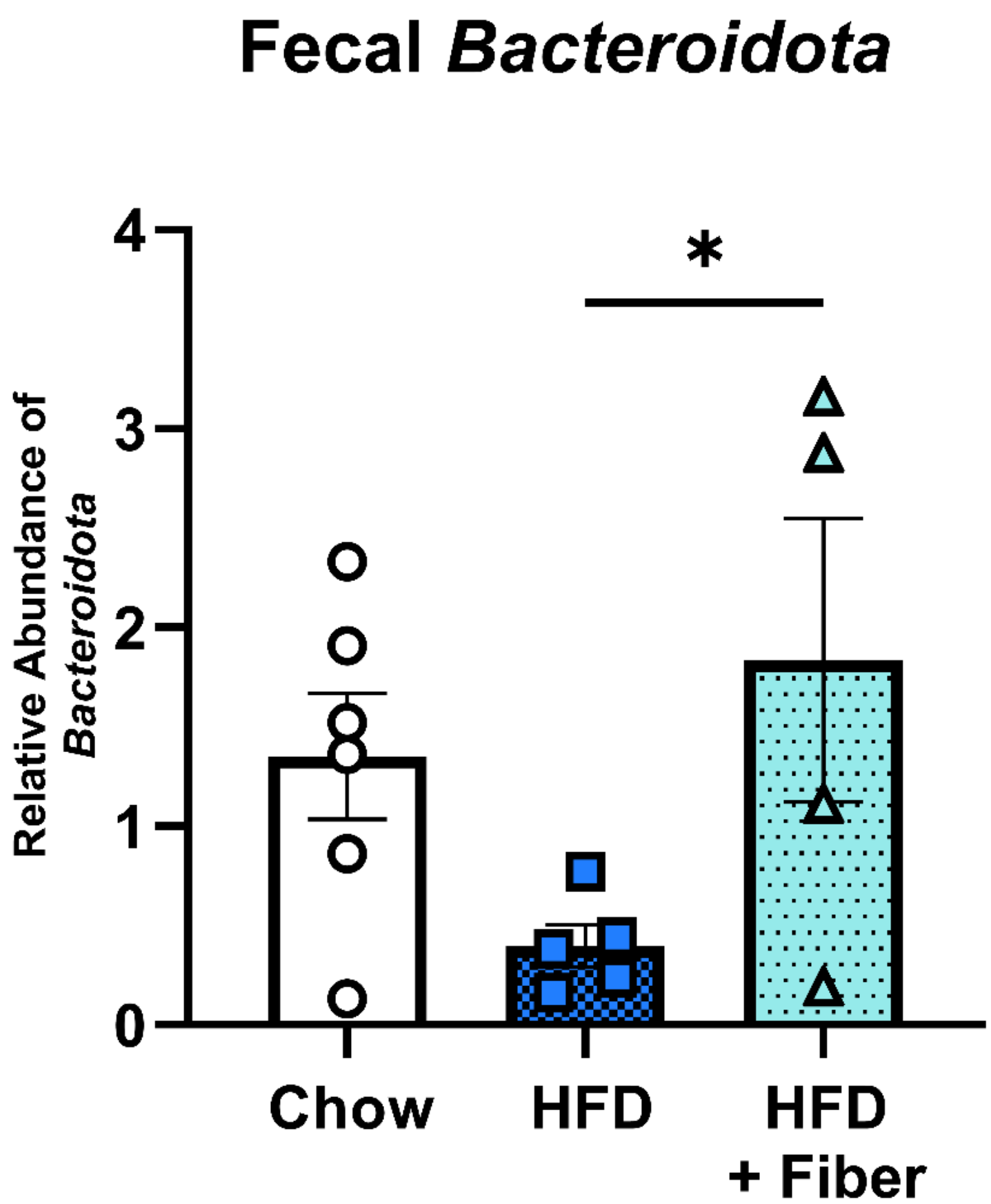

Fig. S1. Fermentable fiber increases fecal *Bacteroidota* relative abundance. Fecal *Bacteroidota* was evaluated by qPCR using target specific primers as described in the Material and Methods section. Values are mean ± SEM (n = 4-6 per group). Statistical significance determined by one-way ANOVA with Fisher's LSD for multiple comparisons (*P < 0.05).

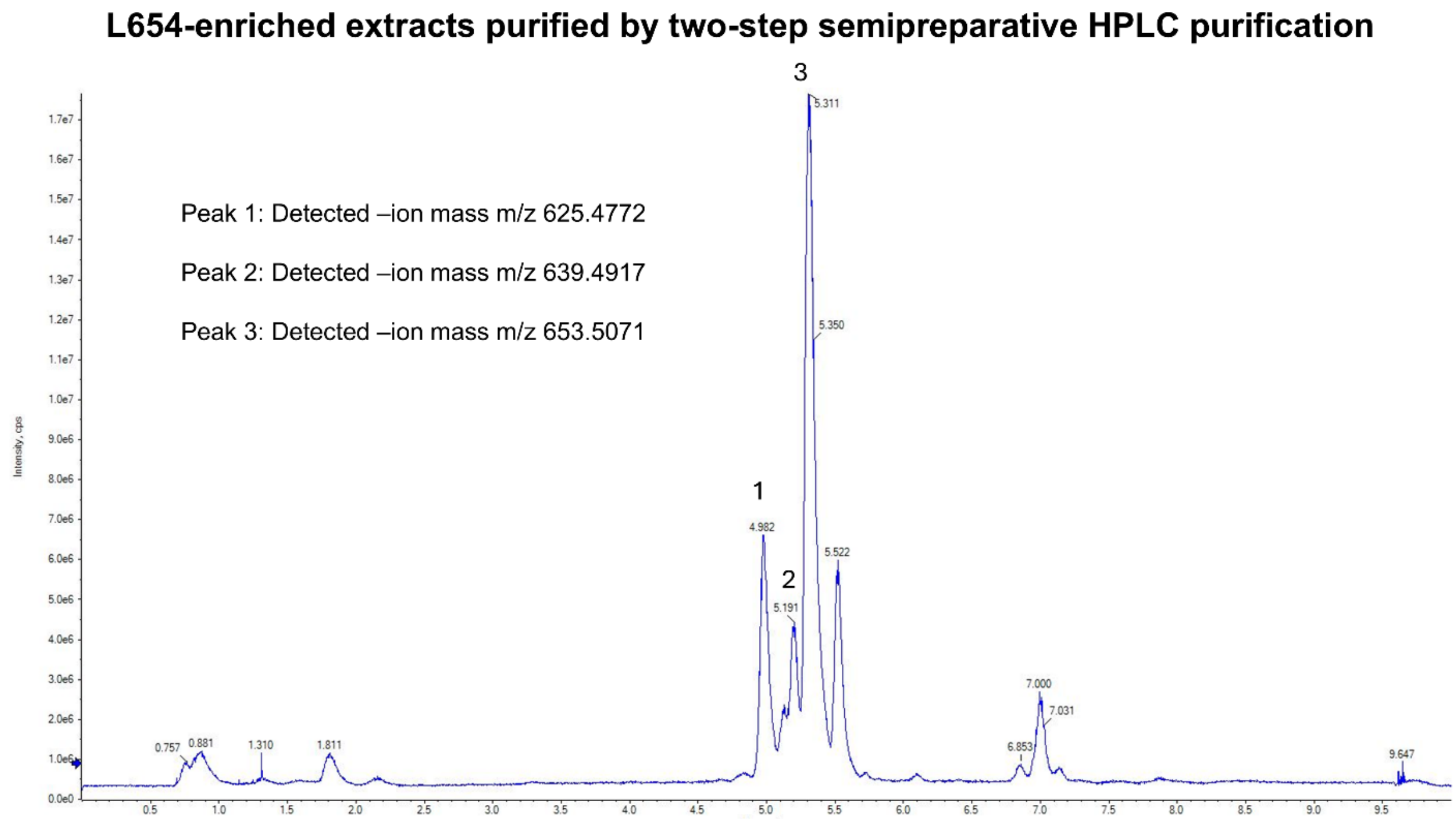

Fig. S2. Purified Lipid 654 extract used for experiments. Bacterial lipids were purified by two-step semipreparative HPLC methods as described in the Materials and Methods section. Detected negative ion *m/z* values of L654 species are shown for the major peaks, with peak 3 coming from the dominant species. The lipid emerging from the minor peak at 5.522 min. is L567.

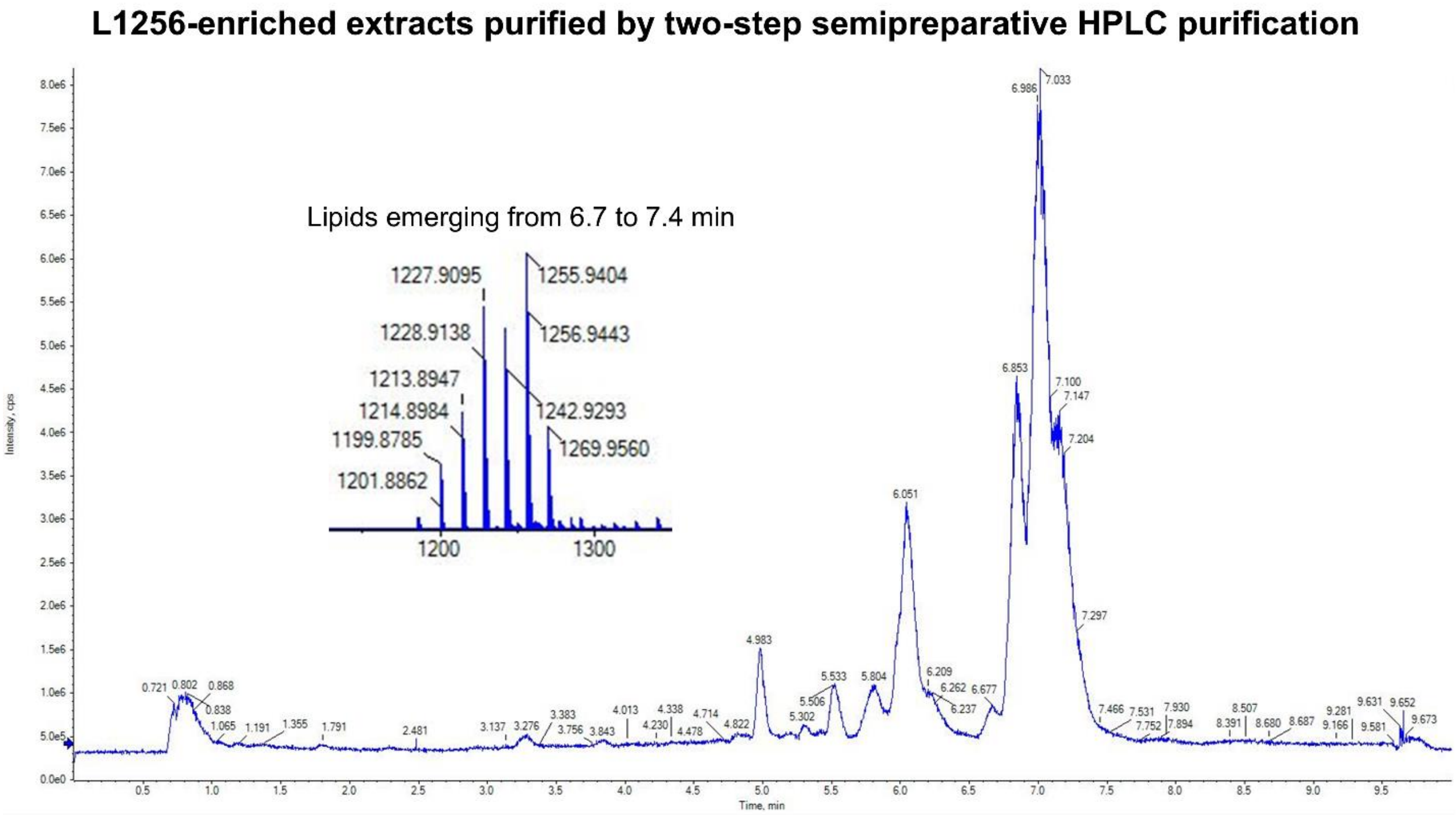

Fig. S3. Purified Lipid 1256 extract used for experiments. Bacterial lipids were purified by two-step semipreparative HPLC methods as described in the Materials and Methods section. Detected negative ion *m/z* values are shown for the major L1256 species emerging from 6.7 to 7.4 minutes, with the dominant ion as *m/z* 1255.9404. The lipids emerging from the minor peak at 6.051 min. are de-esterified L1256 species.

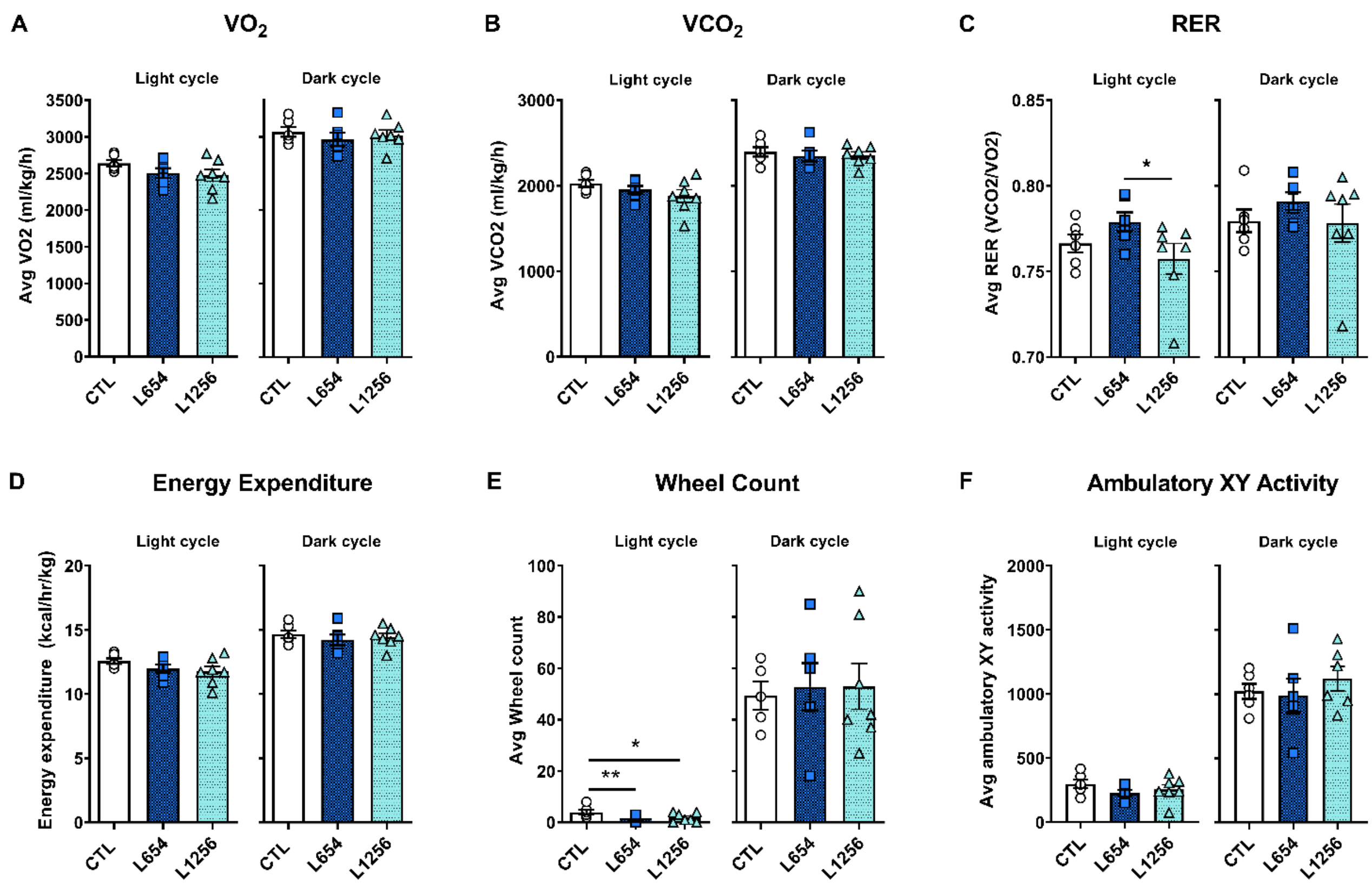

Fig. S4. Limited effects of microbiome glycine lipids on indirect calorimetry measurements. Mice were individually housed in the Oxymax comprehensive laboratory animal monitoring system (CLAMS) cages for three days to measure activity and energy expenditure. After 48 h of acclimation, measurements were recorded hourly for the final 24 h during light cycle and dark cycle phases to determine VO_2 consumed (A), VCO_2 expired (B), respiratory exchange ratio (RER) (C), energy expenditure via heat production (D), wheel count (E), and ambulatory activity (F). Values are mean ± SEM (n = 5-6 per group). Statistical significance determined by one-way ANOVA with Fisher's LSD for multiple comparisons (*P < 0.05, **P < 0.01).

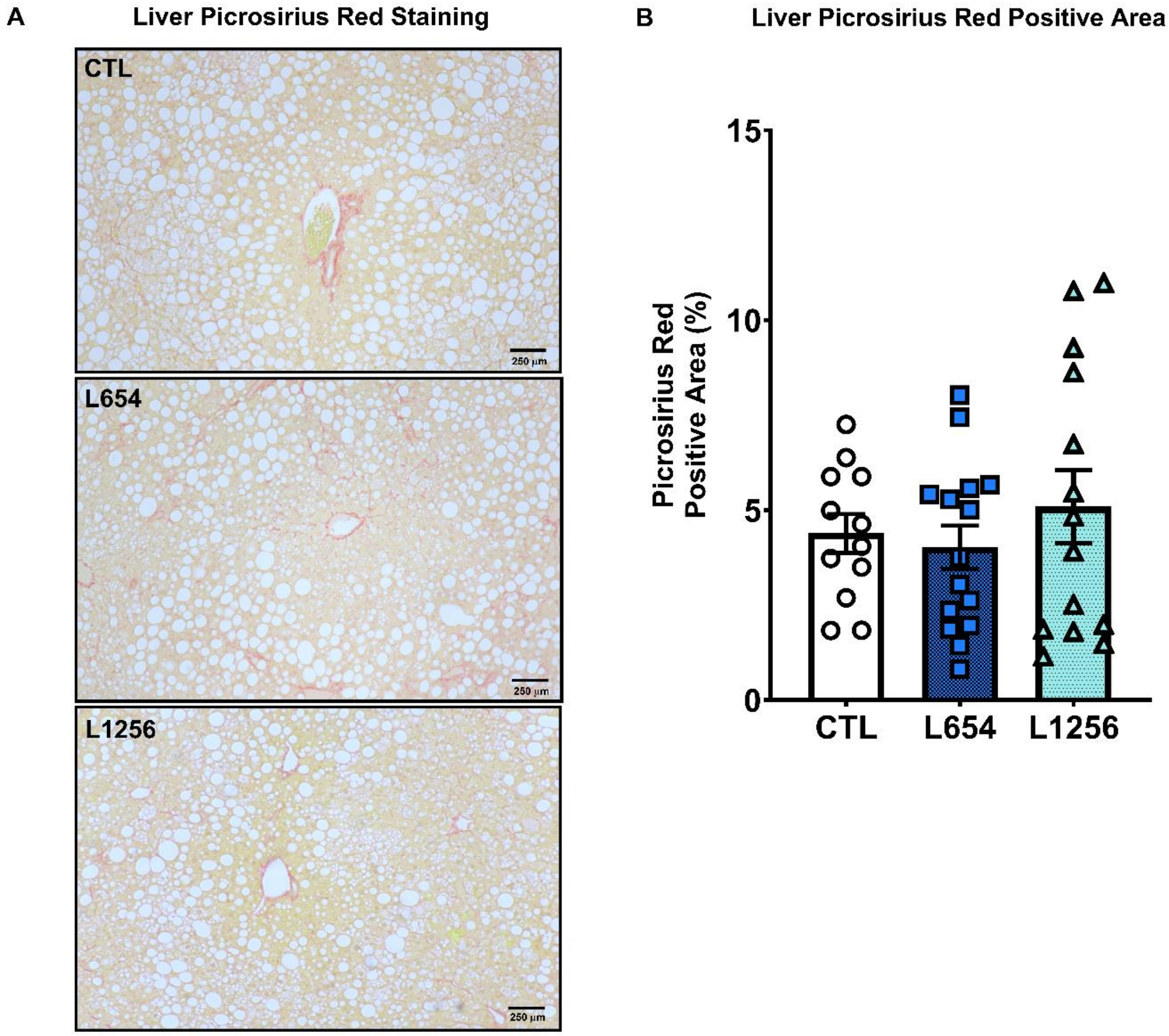

Fig. S5. Microbiome glycine lipids did not affect quantification of fibrotic areas in liver sections. The Livers were formalin-fixed, sectioned, and stained with Picrosirius red to visualize collagen deposition (x200). (A) Representative images of Picrosirius red-stained liver sections from male mice to visualize collagen deposition. (B) Quantification of fibrotic areas in liver sections. (*n* = 13-15, mean ± SEM).

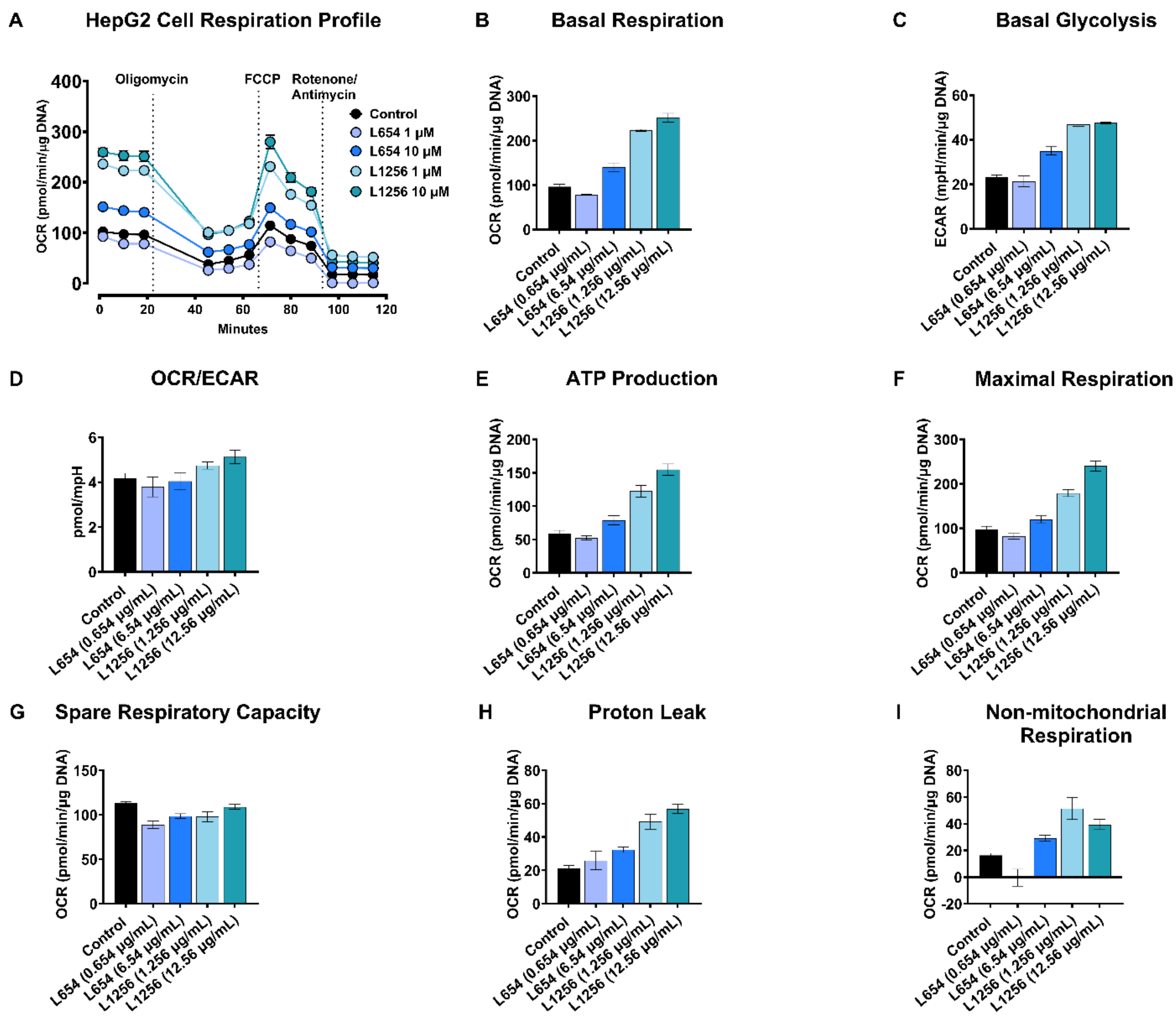

Fig. S6. Representative Mito Stress test experiment in HepG2 cells. HepG2 cells were treated with either bacterial lipid extract preparations enriched in L654 (0.654 μg/mL, 6.54 μg/mL) or L1256 (1.256 μg/mL, 12.56 μg/mL), corresponding to approximately equimolar concentrations (~1 μM and ~10 μM) of the dominant lipid species (i.e., L654 and L1256, respectively), or vehicle control for 72 hours before analysis with the Seahorse XFe24 Extracellular Flux Analyzer using the Mito Stress test. Data from a representative experiment is shown. Mitochondrial respiratory parameters are shown for oxygen consumption rate (OCR) during the Mito Stress test over time (A), basal respiration (B), basal glycolysis

(C), OCR/extracellular acidification rate (ECAR) ratio (D), ATP production (E), maximal respiration (F), spare respiratory capacity (G), proton leak (H), and non-mitochondrial respiration (I).

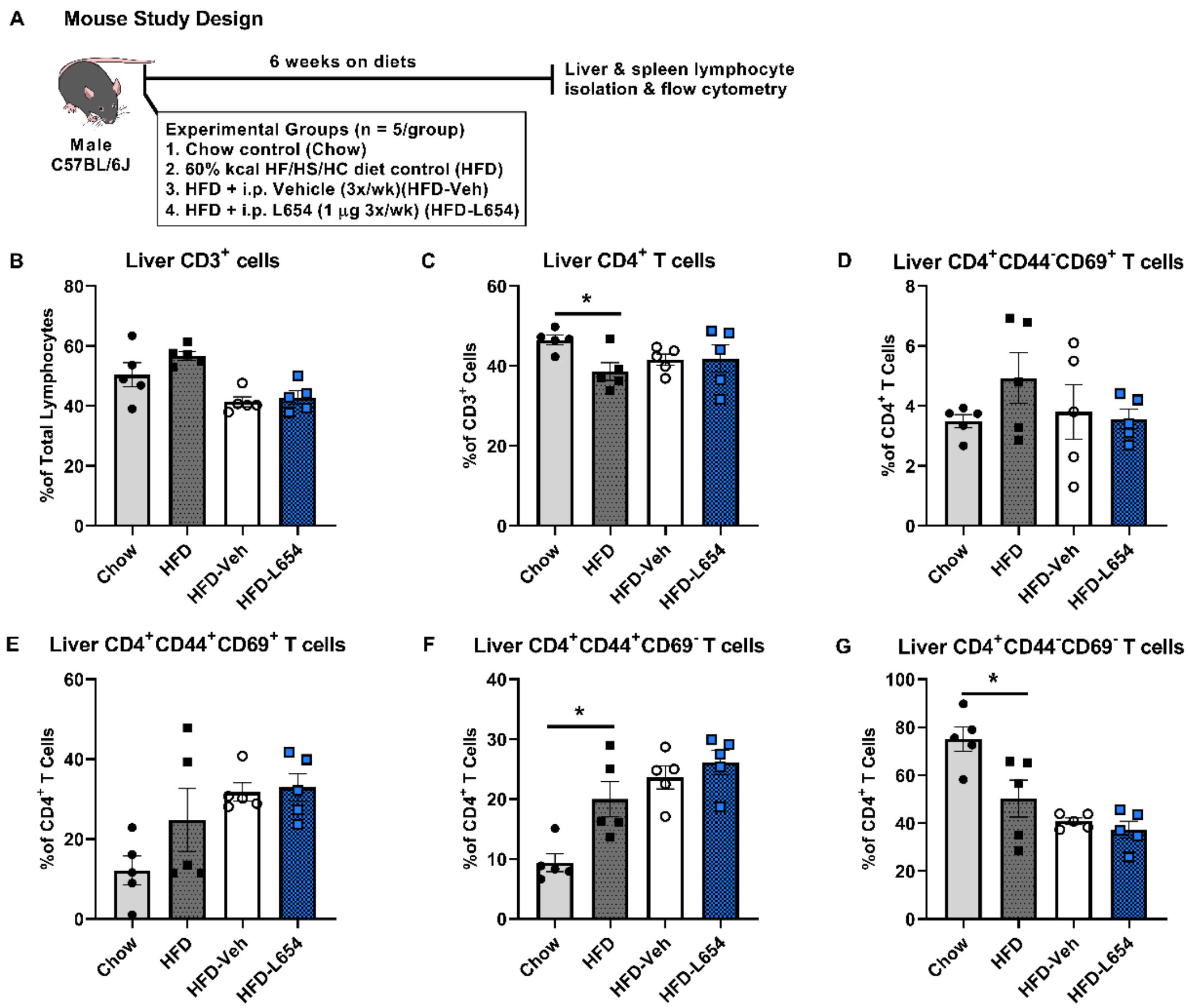

Fig. S7. Lipid 654 does not affect HFD-induced increase in effector/memory liver CD4⁺ T cells and decrease in naïve liver CD4⁺ T cell frequencies. The effects of chronic L654 administration on T cells were studied in mice (A). Male C57BL/6J mice were fed for 6 weeks with one of the following dietary regimens: (1) a standard chow diet (Chow), (2) a high-fat/high-cholesterol/high-sucrose diet (HFD), (3) the HFD with vehicle control intraperitoneal (i.p.) injections (three times weekly; HFD-Veh), or (4) the HFD with i.p. injections of L654-enriched bacterial lipid extract (1 µg/dose, administered three times weekly; HFD-L654). Liver and spleen lymphocytes were isolated for flow cytometry analyses as described in Methods. Lymphocyte frequencies in liver are shown for $CD3^+$ cells (B), $CD4^+$ T cells (C), $CD4^+CD44^-CD69^+$ T cells (D), $CD4^+CD44^+CD69^+$ T cells (E), $CD4^+CD44^+CD69^-$ T cells (F), $CD4^+CD44^-CD69^-$ T cells (G). Values are mean ± SEM ($n = 5$ per group). Statistical significance determined by one-way ANOVA with Fisher's LSD for multiple comparisons (*$P < 0.05$, **$P < 0.01$).

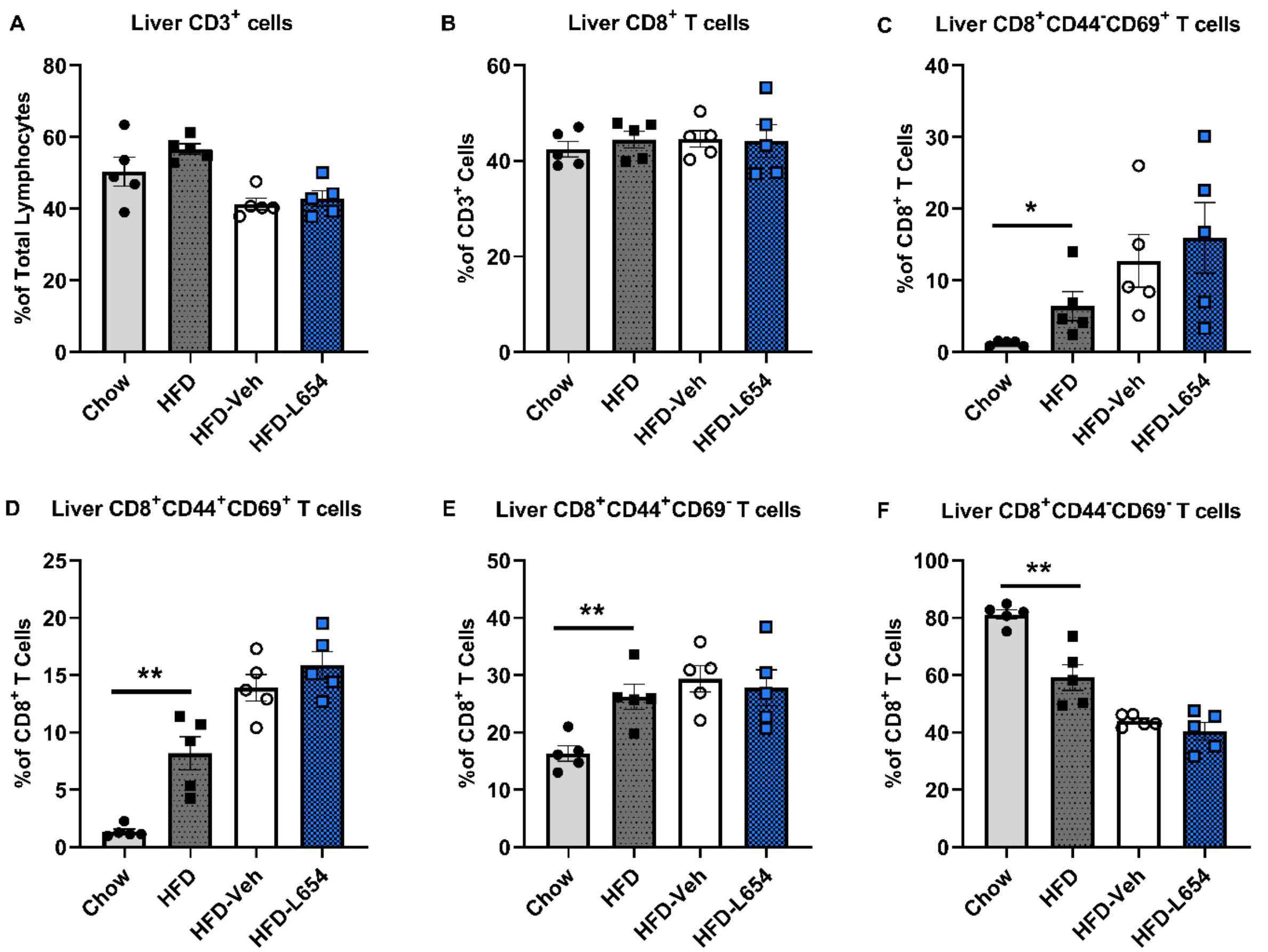

Fig. S8. Lipid 654 does not affect HFD-induced increase in activated/memory liver CD8$^+$ T cells and decrease in naïve liver CD8$^+$ T cell frequencies. Lymphocyte frequencies in liver are shown for CD3$^+$ cells (A), CD8$^+$ T cells (B), CD8$^+$CD44$^-$CD69$^+$ T cells (C), CD8$^+$CD44$^+$CD69$^+$ T cells (D), CD8$^+$CD44$^+$CD69$^-$ T cells (E), CD8$^+$CD44$^-$CD69$^-$ T cells (F). Values are mean ± SEM (*n* = 5 per group). Statistical significance determined by one-way ANOVA with Fisher's LSD for multiple comparisons (*P < 0.05, **P < 0.01).

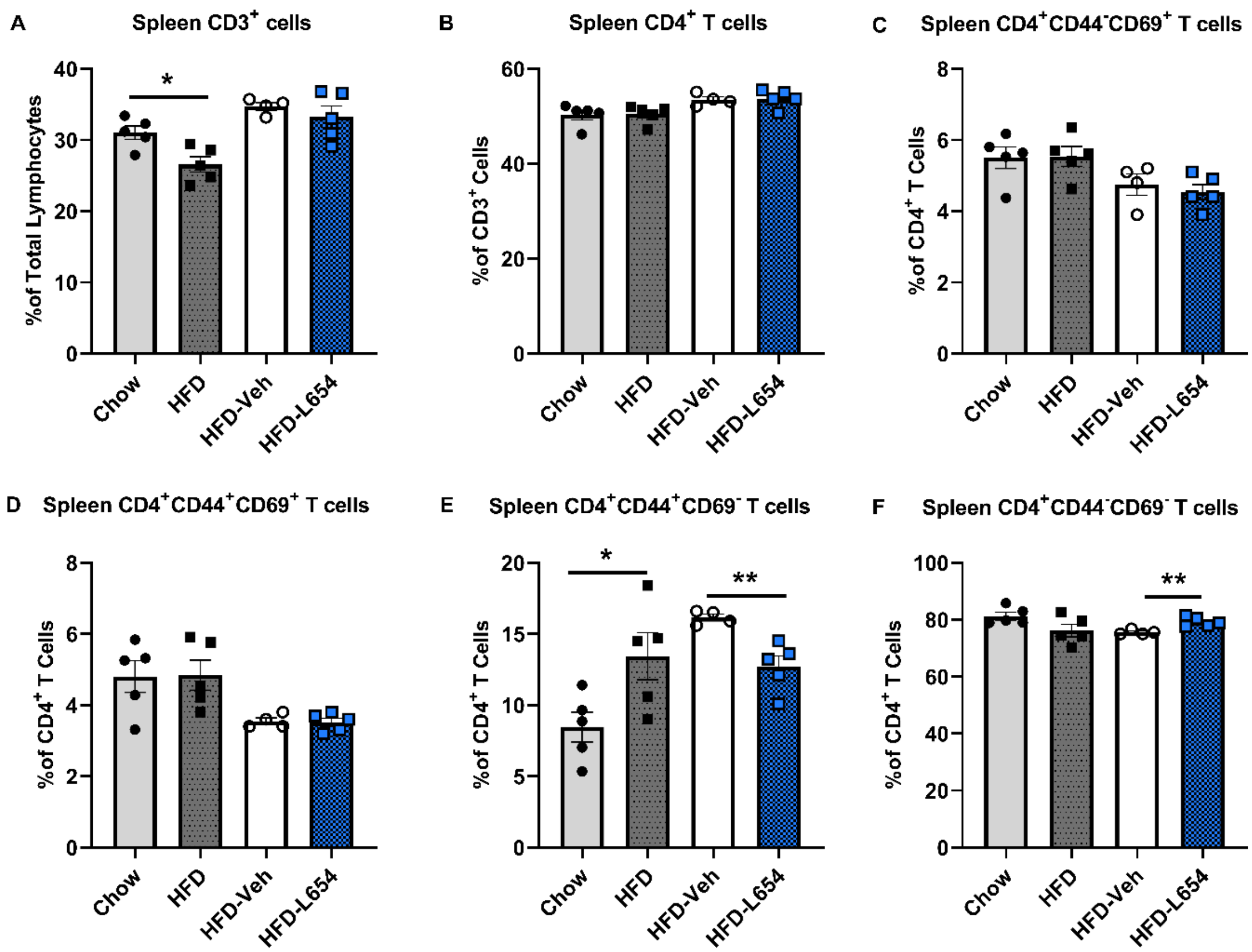

Fig. S9. Lipid 654 decreases the frequency of effector/memory $CD4^+CD44^+CD69^-$ T cells and increases naïve $CD4^+$ T cells in spleen. Lymphocyte frequencies in spleen are shown for $CD3^+$ cells (A), $CD4^+$ T cells (B), $CD4^+CD44^-CD69^+$ T cells (C), $CD4^+CD44^+CD69^+$ T cells (D), $CD4^+CD44^+CD69^-$ T cells (E), $CD4^+CD44^-CD69^-$ T cells (F). Values are mean ± SEM (n = 5 per group). Statistical significance determined by one-way ANOVA with Fisher's LSD for multiple comparisons (*P < 0.05, **P < 0.01).

Table S1. Composition of Western-type high-fat diets

Diet Component (per 1 kg)	HFD (g)	HFD + Fiber (g)
Casein	195	189
L-Cystine	3	3
Sucrose	340	340
Corn Starch	55.5	11.5
Maltodextrin	60	60
Anhydrous Milkfat	210	210
Soybean Oil	20	20
Cellulose	50	0
Mineral Mix, AIN-93G-MX (94046)	43	43
Vitamin Mix, AIN-93-VX (94047)	19	19
Choline Bitartrate	3	3
TBHQ, antioxidant	0.04	0.04
Cholesterol*	1.5	1.5
Citrus peel pectin (Sigma-Aldrich, P9135)#	0	50
Pea fiber (Jianyuan, HYPF-B3)†	0	50

*Milk fat contributes 0.05% (w/w) cholesterol, resulting in 0.2% total cholesterol (w/w) in diets#Consists of 82% galacturonic acid (dried basis)

†Consists of 61.5% dietary fiber, 12.5% protein, 82.5% carbohydrate, 1% fat, 2% moisture, 2% ash (dried basis)

Table S2. B**ody weights and food intake of mice after 8 weeks of diets**

**Variable**	**Chow**	**HFD**	**HFD + Fiber**
Body weight (g)	27.9 ± 0.49	28.2 ± 0.43	29.6 ± 0.73*
Weight change from baseline (%)	18.0 ± 1.11	30.2 ± 7.00*	27.0 ± 3.93
Food intake (g/day)	3.52 ± 0.18	2.60 ± 0.10*	2.78 ± 0.05*
Liver weight (g)	1.13 ± 0.04	1.30 ± 0.04*	1.37 ± 0.13*

Data are presented as mean ± SEM (*n* = 6–9 per group), except for food intake where *n* = 3-4 per group. *****indicates *p*-value < 0.05 vs. Chow group via one-way ANOVA.

Table S3. **Body and tissue weights of mice after 22 weeks of diet with 8 weeks of treatments in MASH model**

**Variable**	**Control**	**L654**	**L1256**
Body weight (g)	50.6 ± 1.11	50.2 ± 1.39	51.3 ± 1.09
Liver weight (g)	7.75 ± 0.27	7.57 ± 0.35	7.40 ± 0.34
% Liver	3.94 ± 0.21	3.86 ± 0.28	3.66 ± 0.27
Epidydimal adipose weight (g)	1.97 ± 0.12	1.98 ± 0.2	1.95 ± 0.13
% Epidydimal adipose	3.92 ± 0.25	4.08 ± 0.47	3.87 ± 0.30
Total lean mass (g)	31.7 ± 0.63	32.7 ± 0.51	32.1 ± 0.54
Total fat mass (g)	25.3 ± 1.03	24.5 ± 1.20	24.9 ± 1.14
Total body water (g)	27.2 ± 0.68	28.0 ± 0.50	27.8 ± 0.53
Free body water (g)	0.35 ± 0.07	0.14 ± 0.03*	0.18 ± 0.03*
Food intake (g/day)	2.69 ± 0.06	2.67 ± 0.05	2.63 ± 0.05

Data are presented as mean ± SEM (n = 13–15 per group), except for food intake where n = 5 per group. *****indicates p-value < 0.05 vs. Control group via one-way ANOVA.

Table S4. Serum biochemical analyses after 22 weeks of diet with 8 weeks of treatments

Variable	Control	L654	L1256
Total cholesterol (mg/dL)	255.2 ± 21.6	234.6 ± 18.4	225.4 ± 17.1
HDL-C (mg/dL)	84.4 ± 5.9	81.4 ± 3.9	84.6 ± 4.1
Non-HDL-C (mg/dL)	170.8 ± 17.3	154.3 ± 17.0	134.1 ± 15.6
Triglycerides (mg/dL)	45.9 ± 4.86	37.4 ± 4.63	36.8 ± 2.86
ALT (IU/L)	728.7 ± 73.5	560.3 ± 51.3*	593.8 ± 52.3
PON-1 activity (kU/L)	9.43 ± 0.40	10.74 ± 0.53	10.41 ± 0.48
sTLR2 (ng/mL)	5409.5 ± 619.1	3509.1 ± 349.3*#	4685.3 ± 330.2
CCL2 (pg/mL)	552.2 ± 52.2	483.7 ± 45.4	470.2 ± 57.4

Values are mean ± SEM for all groups (n = 13-15/group). ALT, alanine transaminase; CCL2, C-C motif chemokine ligand 2; PON-1, paraoxonase-1; sTLR2, soluble Toll-like receptor 2. *****indicates p-value < 0.05 vs. Control group via one-way ANOVA. **#**indicates p-value < 0.05 vs. L1256 group.

Table S5. Fecal microbiome glycine lipids of mice after 22 weeks of diet with 8 weeks of treatments

Variable	Control	L654	L1256
Total glycine lipids (ng/g)	201247 ± 25515	132227 ± 23086	212771 ± 60925
L1256 (ng/g)	13860 ± 1675	15157 ± 2563	17368 ± 2855
L654 (ng/g)	64333 ± 9380	57782 ± 5863	59364 ± 12291
L567 (ng/g)	9068 ± 1709	16378 ± 8094	11970 ± 1444
L430 (ng/g)	106410 ± 26319	35616 ± 19252	116606 ± 53587
L342 (ng/g)	7575 ± 569	7295 ± 940	7464 ± 976

Values are mean ± SEM for all groups (*n* = 5/group).

Table S6. Calculated and measured masses of lipid standards and synthetic glycine lipids by mass spectrometry

Lipid type	Lipid species	Exact mass	Detected mass
**Synthetic glycine lipids**	Synthetic L567	566.479	566.4776
	Synthetic L430	429.297	429.2948
	Synthetic L342	342.265	342.2633
	Synthetic L654	653.511	653.5057
	L654-d_9	662.5675	662.5639
***P. gingivalis* L654 standard**	L654	653.511	653.5071
	L640	639.4954	639.4917
	L626	625.4797	625.4772
***P. gingivalis* L1256 standard**	L1284	1283.9735	1283.9697
	L1270	1269.9578	1269.9529
	L1256	1255.9422	1255.9373
	L1242	1241.9265	1241.9225
	L1228	1227.9109	1227.9068
	L1214	1213.8952	1213.8923

Table S7. Mass spectrometry multiple reaction monitoring (MRM) parameters

Lipid species	Precursor ion (Da)	Fragment ion (Da)
L342	342.3	342.263
L430	430.3	430.2378
L567	567.5	241.2042
L654	653.5	241.216
L1242	1241.9	605.444
L1256	1255.9	619.4344

Table S8. Composition of high-fat, high-sucrose, high-cholesterol (HF/HS/HC) diet

Diet Component	(g/kg)
Sucrose	340.0
Casein	200.0
L-Cystine	3.0
Lard	300.0
Soybean oil	40.0
Insoluble fiber (Solka-Floc cellulose)	47.0
AIN-93 mineral mix	38.0
AIN-93 vitamin mix	10.0
Choline bitartrate	2.0
t-Butylhydroquinone	0.008
Cholesterol (2%)	20

Table S9. Primer list for qRT-PCR

Gene	Protein*	5'-Forward Primer-3'	5'-Reverse Primer-3'
Acadl	ACADL	TCTTTTCCTCGGAGCATGACA	GACCTCTCTACTCACTTCTCCAG
Acadm	ACADM	AGGGTTTAGTTTTGAGTTGACGG	CCCCGCTTTTGTCATATTCCG
Acot3	ACOT3	TTGTCTGTCTGCTGAACCCGA	CGGCTGGTCACAAAGGCAA
Acot4	ACOT4	GCCTGTTGGAATACCGAGCC	AATGTCTGGGCCTTTTACCTTTG
Acox1	ACOX1	CCCAAGACCCAAGAGTTCATTC	CAGGCCACCACTTGATGGA
Adam17	ADAM17	AGGACGTAATTGAGCGATTTTGG	TGTTATCTGCCAGAAACTTCCC
Adgre1	ADGRE1	GAGTGGAATGTCAAGATGTTA	CAGTGGAAGAAGAGAAGC
Bcl6	BCL6	GAAAGGCCGGACACCAGTTT	GCCATGTTGTGTTTGCCCAG
Ccl2	CCL2	TTCCTCCACCACCATGCAG	CCAGCCGGCAACTGTGA
Ccn1	CCN1	AGAGGCTTCCTGTCTTTGGC	CCAAGACGTGGTCTGAACGA
Cd74	CD74	CCTTGCTGATGCGTCCAATG	CTGGGTCATGTTGCCGTACT
Col1a1	COL1A1	AGGTATGCTTGATCTGTAT	CAGTCCAGTTCTTCATTG
Col3a1	COL3A1	GAAAGTGAGGGAAGCCAAA	TCAGAGTAGCACCATCAGT
Gapdh	GAPDH	TGTGTCCGTCGTGGATCTGA	CCTGCTTCACCACCTTCTTGAT
H2aa	H2-Aa	GAGGAGCCGGTTCTGAAACA	TCGCAGGCCTTGAATGATGA
H2dmb1	H2-DMb1	ACTGCCTCTATTCAGGTCCCC	CTTGAACCCCCACCTCATTTCA

H2eb1	H2-Eb1	CTGTCACGGTCGAGTGGAAA	CCTGTTGGCTGAAGTCCAGA
Il10	IL-10	GGCGCTGTCATCGATTTCTC	ATGGCCTTGTAGACACCTTGG
Il1b	IL-1β	GTCACAAGAAACCATGGCACAT	GCCCATCAGAGGCAAGGA
Pon1	PON1	ACTGGTGTTGGCACTTTAC	ACTGGTGTTGGCACTTTAC
Ppara	PPARα	CGGCAGTGCCCTGAACA	TGGTACCCTGAGGCCTTGTC
Ppargc1a	PGC1α	AAGCTGAAGCCCTCTTGCAA	ACTGTACCGGGCCCTCTTG
Rplp0	RPLP0	CCTGAAGTGCTCGACATCAC	CCACAGACAATGCCAGGAC
Saa1	SAA1	CCAATTACTACAGACCTCC	AGCATCTTCAGTGTTCCTA
Tgfb1	TGF-β1	CGCAACAACGCCATCTAT	TGCTTCCCGAATGTCTGA
Tlr2	TLR2	CAGTCTTCCTAGGCTGGTGC	AAGGAAACAGTCCGCACCTC
Tlr4	TLR4	CCTGCATAGAGGTAGTTC	TGGTTGAAGAAGGAATGT
Tnf	TNF-α	GGCTGCCCCGACTACGT	ACTTTCTCCTGGTATGAGATAGCAAAT
Trem2	TREM2	CTGGAACCGTCACCATCACTC	CGAAACTCGATGACTCCTCGG

*Abbreviations used: ACADL, Long-chain acyl-CoA dehydrogenase; ACADM, Medium-chain acyl-CoA dehydrogenase; ACOT3, Acyl-CoA thioesterase 3; ACOT4, Acyl-CoA thioesterase 4; ACOX1, Acyl-CoA oxidase 1; ADAM17, ADAM metallopeptidase domain 17; ADGRE1, Adhesion G protein-coupled receptor E1; BCL6, B-cell lymphoma 6; CCL2, C-C motif chemokine ligand 2; CCN1, Cellular communication network factor 1; CD74, CD74 molecule (invariant chain of MHC class II); COL1A1, Collagen type I alpha 1 chain; COL3A1, Collagen type III alpha 1 chain; GAPDH, Glyceraldehyde-3-phosphate dehydrogenase; H2-Aa, Histocompatibility 2, class II antigen A, alpha; H2-DMb1,

Histocompatibility 2, class II antigen M beta 1 chain; H2-Eb1, Histocompatibility 2, class II antigen E beta; IL-10, Interleukin-10; IL-1β, Interleukin-1 beta; PON1, Paraoxonase 1; PPARα, Peroxisome proliferator-activated receptor alpha; PGC1α, PPAR gamma coactivator 1-alpha; RPLP0, Ribosomal protein lateral stalk subunit P0; SAA1, Serum amyloid A1; TGF-β1, Transforming growth factor beta-1; TLR2, Toll-like receptor 2; TLR4, Toll-like receptor 4; TNF-α, Tumor necrosis factor; TREM2, Triggering receptor expressed on myeloid cells 2.

Table S10. Primer list for mitochondrial and nuclear DNA analysis

Gene	Species	Location	Product	5'-Forward Primer-3'	5'-Reverse Primer-3'
Hk2	Mouse	Nucleus	Hexokinase-2	GCCAGCCTCTCCTGATTTTAGTGT	GGGAACACAAAAGACCTCTTCTGG
HK2	Human	Nucleus	Hexokinase-2	AAGACATTAGAGCATCTG	CTCCATTTCTACCTTCAT
LPL	Human	Nucleus	Lipoprotein lipase	CGAGTCGTCTTTCTCCTGATGAT	TTCTGGATTCCAATGCTTCGA
MT-CYB	Human	Mitochondria	Cytochrome b	CCCTAGCCAACCCCTTAAACA	GGACGGATCGGAGAATTGTG
MT-ND1	Human	Mitochondria	NADH dehydrogenase 1	CCATCACCCTCTACATCACCGCCC	GAGCGATGGTGAGAGCTAAGGT
Mt-Rnr2	Mouse	Mitochondria	16S ribosomal RNA	CCGCAAGGGAAAGATGAAAGAC	TCGTTTGGTTTCGGGGTTTC
Rn18s	Mouse	Nucleus	18S ribosomal RNA	CGCGGTTCTATTTTGTTGGT	AGTCGGCATCGTTTATGGTC
